# Characteristics and Cryopreservation of Semen of Sex-Reversed Females of Salmonid Fish

**DOI:** 10.3390/ijms22020964

**Published:** 2021-01-19

**Authors:** Sylwia Judycka, Joanna Nynca, Piotr Hliwa, Andrzej Ciereszko

**Affiliations:** 1 Department of Gametes and Embryo Biology, Institute of Animal Reproduction and Food Research, Polish Academy of Sciences, Tuwima 10, 10-748 Olsztyn, Poland; j.nynca@pan.olsztyn.pl (J.N.); a.ciereszko@pan.olsztyn.pl (A.C.); 2 Department of Ichthyology and Aquaculture, University of Warmia and Mazury in Olsztyn, Warszawska St. 117A, 10-701 Olsztyn, Poland; phliwa@uwm.edu.pl

**Keywords:** cryopreservation, masculinisation, maturation, salmonids, sex-reversed females, sperm quality

## Abstract

Sex reversal has been used as a breeding strategy by salmonid fish to produce genetically and phenotypically single sex populations. Production of all-female fish has great importance for the creation of monosex female triploids of salmonid fish, which are valued for their sterility, lack of female maturation, and larger commercial size. Among salmonids, the majority of rainbow trout (*Oncorhynchus mykiss*) production is based on all-female production with a high proportion of all-female triploid production in Europe. The main aim of this review is to present the recent knowledge regarding sex-reversed females (SRFs) of salmonid fish. We discuss the methods of sex reversal as well as their effects on the morphology and histology of the reproductive tract. We focus on the characteristics of SRF semen as well as the factors determining semen quality. The lower quality of SRF sperm compared to that of normal males has resulted in the need for the artificial maturation of semen. Most importantly, methods of semen storage—both short-term and long-term (cryopreservation)—that can improve hatchery operations are presented with the special emphasis on recent progress in development of efficient cryopreservation procedures and use of cryopreserved semen in hatchery practice. Moreover, we also address the emerging knowledge concerning the proteomic investigations of salmonid sperm, focusing primarily on the proteomic comparison of normal male and SRF testicular semen and presenting changes in SRF rainbow trout sperm proteome after in vitro incubation in artificial seminal plasma.

## 1. Introduction

### 1.1. Definition

**Figure 1:**
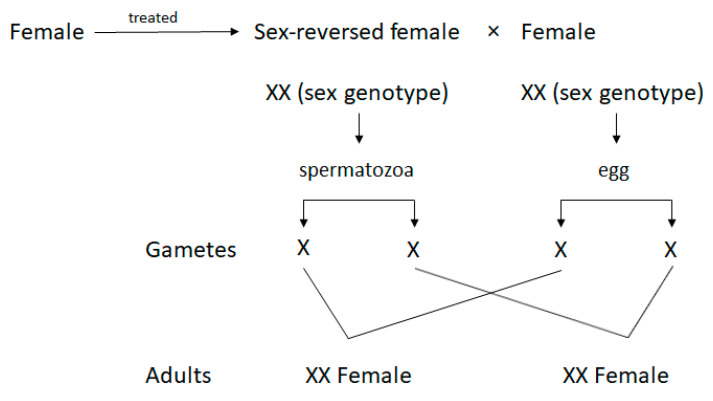
Mating of sex-reversed female with normal female, showing all-female fish.

Sex-reversed females (SRFs; masculinised females, neomales) are genetically females; however, owing to masculinisation, they are capable of producing spermatozoa [1]. Females are masculinised by the hormonal treatment of fertilised eggs or larvae with androgens or their analogues before sex differentiation [2]. However, SRFs usually have less developed testes and there is typically a lack of spermatic ducts [3]. Therefore, it is necessary to sacrifice the fish to collect the semen. The sex determination model of salmonid fish is of the XY type; due to masculinisation, the spermatozoa produced in the testes of SRF possess an X chromosome [3]. Therefore, SRF semen is purely used in the creation of all-female stocks when eggs are fertilised using an X-chromosome-bearing spermatozoon [4] (Figure 1).

### 1.2. The Use of SRFs in Aquaculture

The formation of female monoculture stocks of salmonids is of interest in commercial aquaculture because of its several advantages [1,2,5,6]. It reduces early maturation and limits the number of broodstock fish required [7]. Moreover, since salmonid females reach their commercial size before becoming sexually mature, the nourishment is used only for somatic growth and not for the development of the reproductive system [1]. The low quality of male meat, for example, that of Atlantic salmon (*Salmo salar*), has also been indicated [8]. The production of all-female fish also has great importance for the creation of monosex female triploids of salmonid fish, which are valued for their sterility, lack of female maturation, and larger commercial size [1,9]. Nowadays, among salmonids, the majority of rainbow trout (*Oncorhynchus mykiss*) production is based on all-female stocks, with a high proportion of all-female triploid fish occurring in Europe [10].

### 1.3. Justification

To the best of our knowledge, the sex reversal of salmonid fish has previously been discussed by several excellent reviews [2,11,12,13]. Those reviews mainly focused on the sex control and hormonal induction of sex reversal in fish. The present review distinguishes itself from the mentioned previous studies by focusing on presenting the more recent knowledge concerning salmonid fish SRFs. We discuss the methods of sex reversal and its effect on the morphology and histology of the reproductive system. We focus on the characteristics of SRF semen, as well as the variability of its quality resulting in a need for artificial semen maturation. Most importantly, the methods of semen storage—both short-term and long-term (cryopreservation)—that can improve hatchery operations are presented with the special emphasis on recent progress in development of efficient cryopreservation procedures and use of cryopreserved semen in hatchery practice. Moreover, we also address the emerging knowledge concerning the proteomic investigations of salmonid sperm, focusing primarily on the proteomic comparison of normal male and SRF testicular semen and presenting changes in SRF rainbow trout sperm proteome after in vitro incubation in artificial seminal plasma.

## 2. Hormonal Induction of Sex Reversal

### 2.1. Historic

Sexual development in gonochoristic teleosts is protracted and plastic, which makes it possible to reverse sex both toward the masculinisation of females and feminisation of males [2,13,14]. The use of synthetic hormones to sex reverse salmon was initiated as early as 1937 [2,15]. The masculinisation of Salmo sp. with testosterone or its analogue was conducted in the 1950s [11,16]. Major progress in female masculinisation leading to the implementation of this technique into hatchery practice was achieved in 1970 for several salmonid species, such as rainbow trout, salmon, coho salmon (*Oncorhynchus kisutch*), and chinook salmon (*Oncorhynchus tshawytscha*) [12].

### 2.2. Masculinisation—Type of Hormones

There are two potential approaches for the production of monosex female groups of fish: Direct, based on the use of oestrogens to feminise genetic males [17] and indirect, based on the use of androgens to induce the sex reversal of homogametic females and their subsequent use as broodstock with the use of their spermatozoa to fertilise ova from untreated females, which results in the production of 100% female progenies [18]. The method for indirect feminisation appears to be very efficient for homogametic female fish species, which is characteristic for salmonids [19]. Therefore, indirect feminisation was found to be superior and is now a commonly used in hatchery practice. Major androgens that are being used for female masculinisation include synthetic 17 α-methyltestosterone (MT) and 17 α-methyldihydrotestosterone (MDHT), which represent aromatisable and nonaromatisable androgens, respectively [20,21]. Another important androgen used under practical hatchery conditions is naturally occurring 17 β-hydroxyandrostenedione (OHA). OHA was found to be superior to MT in comparative studies [22,23]. It was also recommended to select OHA if functional males are desired [24,25].

It has to be underlined that due to the commercial use of sex reversal, the detailed methods used to produce SRFs are proprietary to the producers and not available publicly, which restricts our knowledge (to an unknown extent) regarding the usefulness of specific protocols [26].

### 2.3. Important Factors for Sex Reversal

There are a few major factors that need to be considered for the sex reversal of particular salmonid species: (i) The timing of sexual differentiation, (ii) methods of steroid administration and their dose, and (iii) species-specific characteristics important for the efficacy of sex reversal. The multi-factorial determination of sex reversal efficacy makes its control difficult and leads to different extents of male reproductive system development, such as sterile, bisexual, dysfunctional, and normal males [23] (Table 1). A more detailed classification was proposed [6], where sterile or immature males or females, males spermiating or females ready to ovulate, males or females in development, males without functional ducts, intersex, males spermiating (one testis), females ovulating (one ovary), and undifferentiated individuals were distinguished.

The key steps for optimising the production of all-female populations are (a) determining the developmental stage at which gonadal differentiation occurs, (b) optimising the dose and duration of treatment with masculinising agents, and (c) distinguishing SRFs from normal males for breeding purposes.

### 2.4. Timing of Sexual Differentiation

The timing of sexual differentiation during ontogenic development is critical for the advancement of the sex reversal procedure because the efficacy of steroids is at its highest while it is administrated during the so called “labile period”, i.e., starting when the gonads are undifferentiated and continuing through gonadal differentiation. Any deviations from the labile period decreases the efficacy of sex reversal and elevated doses of hormones and longer application times may be required to counteract these losses [13]. Although sexual differentiation mostly starts around the time of hatching, some species-specific differences do occur; for example, in brook trout (*Salvelinus fontinalis*), the pre-hatch period was found as a potential target period for sex reversal using androgen treatment [29]. Moreover, the temperature around the time of hatching has an important modulation effect because embryo development is temperature-dependent; this is being used in hatchery practices to control the time of hatching and growth of larvae. For some rainbow trout strains, such as “Mal” populations, high temperatures alone (up to 18 °C) can induce masculinisation [30]. To compensate for the temperature factor commonly used in fish breeding, degree-days (usually °C-days) are often used for the definition of hormone administration (see Table 2).

### 2.5. Methods of Steroid Administration and Their Doses

There are three methods for steroid administration: (i) Injection (either intramuscular or intraperitoneal), (ii) immersion in a static bath, and (iii) hormone supplementation in the diet. The injection of embryos or larvae is clearly not practical; consequently, the latter two methods are being adopted in hatchery practices, both separately and in combination (Table 2). The selection of the applied method is often clearly related to the labile period for particular species; immersion is preferred when sexual differentiation occurs during the embryonic or larval stages, whereas the dietary treatment is suited for species with differentiation during and/or after the initiation of larval feeding [13]. It also needs to be considered that steroids concentrations may decrease with storage time, especially at room temperature [21,40]. Optimising the dose and duration of treatment with masculinising agents is of the utmost importance; too-low concentrations may be ineffective (however, it may be useful for the production of functional males [38]), whereas too high doses often have a so called “paradoxical feminisation” or a sterilising effect [1,41,42].

### 2.6. Environmental Pollution Issues Related to Sex Reversal under Hatchery Conditions

In recent years, there has been growing concerns about the contamination of the aquatic environment with water pollutants due to anthropogenic activity, which is a serious threat for the reproduction of aquatic animals [43]. Endocrine disrupting chemicals (EDCs) are especially dangerous owing to their interference with endocrine functions. Synthetic androgens used for sex reversal are potential EDCs and their threat is causing increasing awareness in some countries. Therefore, methods to eliminate or decrease their concentrations in water will need to be developed. It seems clear that protocols based on immersion are potentially easy to control because androgens are confined to a restricted volume of vessels for a short time. On the contrary, feeding is more prone to water pollution with hormones, and lasts a long time (usually 60–80 days) so dramatically more hormones are used; moreover, androgens can leak from the diet to the water.

### 2.7. Species-Specific Characteristics Important for the Efficacy of Sex Reversal

The effective steroid doses for sex inversion were found to vary both between and within salmonid species [31]. Species-specific characteristics important for the efficacy of sex reversal may include the degree of gonadal development at the time of treatment, efficacy and specificity of steroid metabolism, and number and specificity of steroid receptors [13]. For some species, such as rainbow trout, efficient protocols must be developed; however, for others, such as brook trout, the efficacy of the procedure needs to be improved [6,28,32]. An extensive review of earlier protocols for particular species of salmonid fish was provided by other authors [12]. Examples of protocols published later are presented in Table 2.

### 2.8. Identification of SRF

The identification of SRFs is not necessary when genetic sex XX is clearly established either due to gynogenetic fish or the progeny of SRF and, in such a case, protocols aiming to produce functional (i.e., with normal spermatic ducts and strippable) semen are desired [39,44]. The production of functional males and their use for fertilisation is clearly advantageous because males can be used several times for two to three years. The problem occurs when the normal population is subjected to masculinisation and the sampling of genetic males is probable. The practical strategy in such a case is focused on the production of non-functional SRFs with easily identified phenotypes, such as hermaphrodites or males without spermatic ducts [21,45,46]. This would ensure that SRF population have the XX genotype and are not mixed with individuals presenting the XY genotype. To the best of our knowledge, this strategy seems to be the most popular in the production of salmonid fish SRFs under hatchery conditions. Recently, ultrasonography was found to be potentially useful for the identification of SRF rainbow trout [47].

## 3. Morphology and Histology of the Reproductive Tract

**Figure 2:**
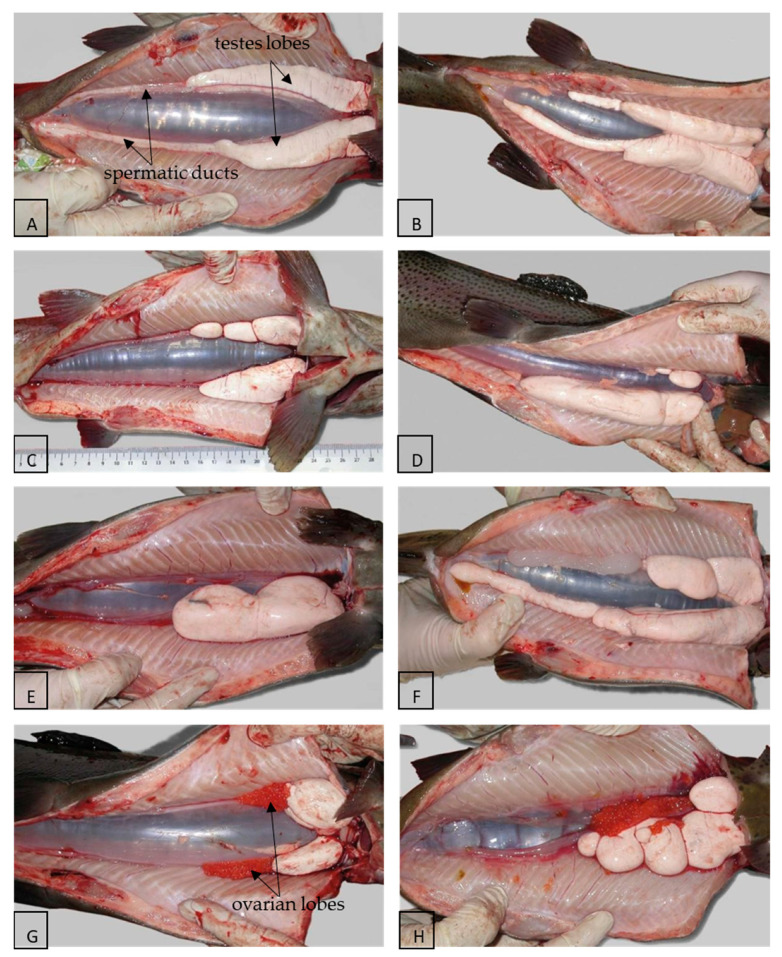
Types of macroscopic and morphological gonadal structures of rainbow trout sex-reversed females: (**A**) twin normal and similar sized testes lobes and functional permeable spermatic ducts; (**B**) twin normal and similar sized testes lobes and dysfunctional occluded spermatic ducts; (**C**) segregation and deep narrowing of one testes lobe; (**D**) clear asymmetry of testes size; (**E**) abnormal single testes lobe without spermatic ducts; (**F**) abnormal asymmetric and constricted testes lobes without spermatic ducts; (**G**) lobular type with a similar sequence of testicular and ovarian lobes on both gonad strands; and (**H**) asymmetric and constricted testes lobes with ovarian tissue within one gonad strand.

The consequences of hormonal treatments used to obtain SRFs are disorders of the anatomical development process of the gonads, which are manifested by the disruption of normal development, including the narrowing, disproportion, or atrophy of the gonadal lobes [1,22,24,48]. Those disturbances can be caused by several factors, including difficulties in the oral administration of hormones at an accurate dose for fishes at the same time as the masculinisation process [49]. Malformations are related mainly to the various stages of development or the lack of spermatic ducts, but also to the macroscopic structure of less well-developed testes lobes [3,50] (Figure 2; Table 1). These malformations are not present in normal males, where two symmetrical and elongated gonadal lobes, each having a well-developed spermatic duct running to the caudal direction and emptying into the genital papilla, are observed [3,47].

**Figure 3:**
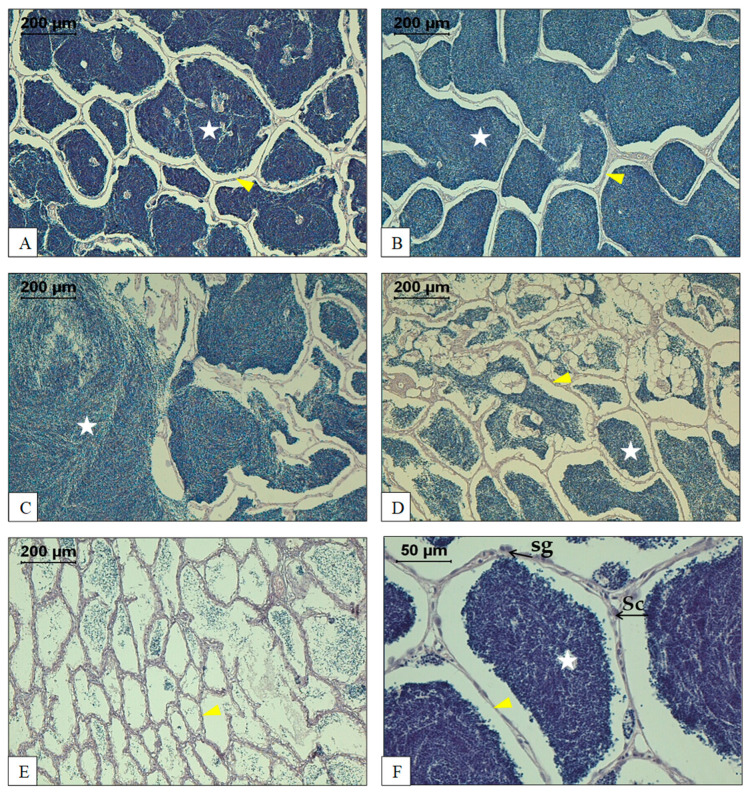
Histological cross-sections of sex-reversed female (SRF) rainbow trout gonads during the spawning season: (**A**) Similar-sized seminiferous tubules at the beginning of the spawning season filled by spermatids and spermatozoa. Ampullae walls separated from the central parts of structures by space isolated from storage within cytological elements; (**B**) tubules with different spatial structures (different sizes), and the walls isolated by the space (gap) between the germ cells located inside the whole tubule structure; (**C**) the unsettled spatial structure of seminiferous tubules during the peak of the spawning season, where part of the walls were perforated and spermatozoa filled most of the available testes space; (**D**) tubules completely or partially free of spermatozoa, which lose their regular shape, and spermatozoa filled all spaces between the interstitial tissue elements, i.e., the walls of the seminiferous tubules; (**E**) seminiferous tubules were mostly empty with an irregular spatial structure at the end of the spawning season. The walls of tubules had increased thickness where a large number of spermatogonia and primary spermatocytes appeared; (**F**) magnification of the single seminiferous tubule filled with spermatids and spermatozoa at the beginning of the spawning season. Description: Seminiferous tubules filled with spermatids and/or spermatozoa (white asterisk); walls of tubules—interstitial tissue (yellow arrowhead); Sc, Sertoli cells; sg, spermatogonia.

Histologically, the testes of mature SRF rainbow trout consist of large lobules (about 0.5–1 mm in diameter) located in the centre and smaller lobules (about 50 μm) situated in the outer parts of the gonad (Figure 3). The thickness of the outer cell wall usually ranges from 10 to 60 μm [49]. Cysts of maturing sperm are visible in the connective tissue of the lobules. Centrilobular cell walls and spermatogonia are frequently seen surrounded by Sertoli cells in the outer parts of the gonads. In the inner parts of the testes, cysts are visibly composed of hypertrophied Sertoli cells, which are not present in the gonads of normal (XY) males [49]. It has been reported that in SRF rainbow trout, the interstitial space became very narrow and poorly developed, and the lumina of the tubules were densely filled with spermatozoa, probably because of the lack of an efferent duct [51]. Additionally, the cysts of epithelial hyperplasia were found. This tissue, filling the lobule, perhaps consists of hypertrophied Sertoli cells, which were also identified in SRF Atlantic salmon [52]. It should be noted that the rate and quality of spermatogenesis are determined by the number and functionality of Sertoli cells present, and therefore, complications with their development can be responsible for pathological processes resulting in reduced semen quality in SRFs [53].

## 4. Semen Characteristics

The masculinisation procedure significantly influences the gonadal development and sperm characteristics of fishes [2]. SRFs have less developed testes and there is usually a lack of spermatic ducts, where the final maturation of spermatozoa occurs [3]. Owing to the lack of spermatic duct secretions, the sperm of SRFs resembles testicular rather than ejaculated sperm and is often characterised by low and highly variable sperm quality (including increased sperm concentration and reduced motility parameters) compared to that of ejaculated sperm from normal males [52,54,55].

### 4.1. Spermatozoa Characteristics

#### 4.1.1. Sperm Concentration

Sperm concentration is one of the major quality parameters that is evaluated as a part of standard fish semen analysis. Unlike in normal males, where sperm concentration is well defined for particular species, in SRFs, two distinct categories of sperm concentration occur—one for non-functional males and the other for functional males. In the case of SRFs, which are often characterised by their lack of spermatic ducts, semen is not diluted by secretions from the spermatic duct and is characterised by high sperm concentrations [54]. The sperm concentration of the testicular semen of SRF varies between species; generally, however, it is within the range of about 20–45 × 10^9^ spermatozoa mL^−1^ (Table 3). For example, for rainbow trout, the average sperm concentration of SRF testicular semen amounts to approximately 30 × 10^9^ spermatozoa mL^−1^, which is about three times higher than the stripped semen of normal males [55,56,57]. Moreover, the sperm concentration values of the testicular semen of SRF are usually similar to values recorded for the testicular semen of normal males [3,49,52]. 

In contrast, as indicated above, masculinisation allows us to obtain fully functional SRFs with developed spermatic ducts, from which semen could be obtained through light manual stripping [7,44]. The stripped semen of SRF coho salmon was characterised by having a similar sperm density to stripped semen of normal males, which amounted to about 20% of the spermatocrit values [7]. Moreover, these values were significantly lower than those of the testicular milt of SRFs (80%). In contrast, it has been found that the spermatocrit values of the stripped semen of SRF Chinook salmon were significantly lower than those of the stripped semen of normal males (about 50% and 75%, respectively) [44].

Often, extremely high sperm concentrations make semen so viscous that it is extremely difficult to sample aliquots with a pipette for sperm concentration measurements, which results in the low accuracy of such measurements. Owing to the high variability in the sperm concentration of especially of non-functional SRFs, the measurement and control of the sperm concentration is extremely important for performing artificial fertilisation and cryopreservation under controlled conditions [69]. The latter is especially important in view of the recently developed standardised method for the semen cryopreservation of SRF based on the controlled number of spermatozoa in straws (see below) [55,58].

#### 4.1.2. Sperm Motility Parameters

Sperm motility is one of the most useful parameters to evaluate fish sperm quality. It allows the prognosis of fertilisation success, as high values of sperm motility parameters generally strongly correlate with fertilisation ability [70]. In the case of semen of SRFs, where spermatozoa do not undergo maturation in spermatic ducts, the sperm motility is almost always lower than that in the semen of normal males [54,55]. The current knowledge concerning sperm motility of testicular semen of SRF is primarily based on the characteristics of SRF rainbow trout, which have been characterised in 19 publications (see Table 3). The sperm motility percentages in SRF Atlantic salmon and brook trout were presented only in single publications and were between 53.0–59.0%, which is similar to that in SRF rainbow trout [52,58]. Moreover, significantly higher values of sperm motility in the testicular semen of normal male Atlantic salmon (81%) than in the testicular semen of SRFs (57%) were recorded [52].

The sperm motility of SRF rainbow trout testicular semen is highly variable and usually within the range of about 18–100%. These values are almost always significantly different from those of normal rainbow trout semen, where the average sperm motility is usually high (within the range of 80–90%) [3,55,60,63]. Moreover, higher percentages of sperm motility in the testicular semen of SRFs than in the testicular semen of normal male rainbow trout were recorded [3,49]. This phenomenon could be caused by the different physiology and morphology of the SRF testis compared to those of the testis of normal males. It should be also noted that the motility duration of SRF rainbow trout spermatozoa lasts up to 40 s and do not differ from values recorded for normal males [3]. However, the motility duration of Atlantic salmon testicular sperm is much longer for normal males (up to 100 s) than for SRFs (up to 67 s; [52]). It must be underlined that information regarding the motility characteristics of SRF semen is often gathered from experiments concerning semen cryopreservation. In such studies, good quality samples are usually selected to secure cryopreservation success. Therefore, percentages of sperm motility can be overestimated compared to those in the original population. The sperm motility of functional SRF males is generally higher than that of non-functional males. At present, data on the sperm motility of functional SRF males are restricted, but the few reports that are available support this suggestion. The sperm motility of functional SRF Chinook salmon was high (about 80%) and did not differ from that of normal males [44]. Similar results were obtained for coho salmon, where the motility of the stripped semen of SRFs was comparable to that of the stripped semen of normal males [7].

Computer-assisted sperm analysis (CASA) is a tool that measures not only the sperm motility, but also sperm velocity parameters (VCL, curvilinear velocity; VSL, straight-line velocity; VAP, average path velocity) and trajectory parameters (LIN, linearity; ALH, amplitude of lateral head displacement; [71]). The values of these parameters differed between species and experiments and are presented in Table 3. However, it is known that different CASA systems might produce differences in measured values [72]. Therefore, the comparison of sperm velocity and trajectory parameters between SRF and normal males is restricted. To the best of our knowledge, a direct comparison between the normal and SRF sperm motility parameters using the CASA system has been performed by just a few authors [52,54,55,60]. Lower VCL values in SRF rainbow trout than in the ejaculated semen of normal males were recorded [55], which indicates that SRF spermatozoa are slower than those of normal males. It has been found that the stripped semen of SRF of coho salmon had similar velocity values to the stripped semen of normal males; however, these values were significantly higher than those recorded for the testicular semen of SRF [7]. Moreover, lower VCL values in the testicular milt of SRF than in that of normal male Atlantic salmon were recorded [52]. The latter also indicated that the VAP, VSL, and ALH values were similar for the testicular semen of SRF and normal males. After dividing semen samples into three classes depending on the sperm motility, it has been found that VCL values are related to the initial quality of sperm and are the highest in the group characterised by >50% sperm motility [56]. Moreover, these values were similar to those obtained for the semen from normal males. It should be considered that the testicular semen of normal males and SRF rainbow trout are characterised by similar sperm motility parameters, which is in accordance with the morphological similarity of both tissues [60]. The testicular spermatozoa of SRFs tend to exhibit lower sperm motility and higher variability in motility parameters compared to those of normal males, which likely is a result of incomplete sperm maturation due to the lack of spermatic ducts.

#### 4.1.3. Sperm Viability

Sperm viability is a key determinant of sperm quality and is mainly based on the analysis of cell membrane integrity and functionality. Sperm viability is often linked to an intact plasma membrane, since the plasmalemma is pivotal for sperm interactions with other cells and their environment [73]. It is notable that the viability was often higher than the sperm motility (see Table 3). The sperm viability values of testicular semen of SRF Atlantic salmon are similar to those of the testicular semen of normal males [52]. The sperm viability of SRF rainbow trout testicular semen is high (above 70%) and does not differ from that of the ejaculated semen of normal males [54,55,60]. Moreover, it has been found that despite differences in sperm motility, the viability of SRF rainbow trout sperm is high and is not dependent on the sperm motility [54]. These results indicated that contrary to the sperm motility, the viability of SRF semen does not seem to be seriously impaired.

#### 4.1.4. Mitochondrial Membrane Integrity and ATP

Mitochondria, located in the sperm midpiece, produce a major part of the ATP essential for sperm motility [74,75]. Damage to the mitochondria may be responsible for the decrease in the percentage of motile spermatozoa as well as for the decrease in spermatozoa ATP levels, both factors being extremely important for sperm functionality. The mitochondrial membrane integrity of SRF rainbow trout spermatozoa was about 68% [67], lower than that of normal males (88–99%; [76,77]). This can explain the differences in sperm motility between SRF and normal males and affirm that sperm motility is primarily dependent on mitochondrial function [78,79].

In contrast, ATP values in SRF rainbow trout semen were found to be high (about 8 nmol ATP/10^19^ cells) and were significantly higher than those recorded for normal male rainbow trout (both testicular and ejaculated semen, about 2.3 and 4.85 nmol ATP/10^19^ cells, respectively; [3]). This suggests that although the energy for sperm movement of SRF spermatozoa seems to be secured, other—unknown at present—disturbances to sperm physiology do not allow this energy to be used for sperm motility.

### 4.2. Seminal Plasma Characteristics

Seminal plasma has a unique composition, containing substances supporting sperm cells and some substances reflecting the function of the reproductive system and spermatozoa [80]. Seminal plasma is an important component of semen that plays a role in sperm metabolism, function, survival, and motility. The main role of seminal plasma in fish is to create an optimal environment for the spermatozoa during the maturation and storage of spermatozoa in the spermatic ducts.

#### 4.2.1. Seminal Plasma Composition

The mineral constituents of seminal plasma have been identified as principal components affecting sperm quality. Their presence or absence interferes with osmolality, pH, and several spermatozoa functions, such as the motility activation of salmonid fish [81]. The five predominant ions in fish seminal plasma are sodium, potassium, chloride, calcium, and magnesium. Knowledge concerning such components in the semen of salmonid SRFs is limited and presented in only one publication [57]. These authors found that concentrations of Na^+^, K^+^, Ca^2+^, Mg^2+^, and Cl^−^ in SRF rainbow trout semen are up to 145.0 ± 20.0, 51.4 ± 7.4, 1.91 ± 0.31, 1.91 ± 0.22, and 161.4 ± 19.2 mM, respectively. Moreover, the concentrations of these ions in SRFs were significantly higher than those in normal males, with the exception of Mg^2+^ (104.2 ± 10.2, 25.2 ± 3.5, 1.24 ± 0.53, 1.91 ± 0.22, and 122.1 ± 11.5 mM for Na^+^, K^+^, Ca^2+^, Mg^2+^, and Cl^−^, respectively). These differences likely reflect the lack of spermatic ducts in SRFs; the spermatic ducts are responsible for the synthesis and secretion of enzymes, monosaccharides, lipids, and proteins and regulate the ionic composition of the seminal fluid [82,83]. Moreover, the differences could be also related to the fact that the sperm hydration phase developed slowly during sperm maturation in the spermatic ducts [84].

#### 4.2.2. Seminal Plasma Osmolality

Seminal plasma osmolality is an important factor responsible for maintaining the spermatozoa in a quiescent state until spawning [85]. The seminal plasma osmolality (about 300 mOsm kg^−1^) reflects the physiological osmolality of body fluids in fish [81]. The seminal plasma osmolality of SRF brook trout semen was more than 300 mOsm kg^−1^, similar to that of SRF rainbow trout, which are within the range of 290–342 mOsm kg^−1^ (Table 3). Moreover, the seminal plasma osmolality values of SRF rainbow trout are almost always higher than those of normal males [54,55,57], which may reflect higher concentrations of mineral and organic compounds in SRFs as well as urine contamination in normal males (see above). It should be considered that the osmolality of the seminal plasma can be considered a reliable marker to detect urine contamination in fish semen [86,87]. Such contamination causes the premature activation of sperm motility due to the low seminal plasma osmolality of urine (and therefore decreased fertilisation ability) and is characteristic for normal males (and presumably functional SRFs) but not non-functional SRFs.

### 4.3. Biochemical Characteristics

#### 4.3.1. Antitrypsin Activity and Protein Concentration

The antitrypsin activity (APA) and protein concentration are important determinants of fish semen quality. The APA of fish seminal plasma is involved in the regulation of spermatogenesis, protection of spermatozoa and reproductive tissues from proteolytic attack, and remodelling of connective tissue of the reproductive system [88,89,90]. Significantly higher APA values in the seminal plasma of SRF rainbow trout (748 ± 416–1453 ± 496 U L^−1^) than in the seminal plasma of normal males (up to 450 U L^−1^) were recorded [54]. High APA values can be related to the greater protection of immature spermatozoa against enzymatic proteolysis in SRF compared to that of normal spermatozoa. The protein concentrations in SRF seminal plasma are generally within the range of 2.9 ± 1.1–8.2 ± 1.3 mg mL^−1^ and are significantly higher in SRFs than in normal males (Table 4; [49,54,56,91]). Moreover, these values are higher in the testicular semen of normal males than in that of SRFs [49]. In contrast, extremely high protein concentrations in SRF rainbow trout semen (27.3 ± 3.0 mg mL^−1^) were recorded [60], which are similar to the testicular semen of normal males. It should also be considered that changes in the APA, which is the major protein in the seminal plasma, correspond to changes in the protein concentration in the fish seminal plasma [54,89].

#### 4.3.2. Lactate Dehydrogenase

Lactate dehydrogenase (LDH) is a spermatozoal enzyme employed as an indicator of injuries that lead to the leakage of LDH from the spermatozoa into the seminal plasma [92]. Consequently, the seminal plasma enzyme concentrations may increase as a result of damage to the sperm membranes. The LDH values in the seminal plasma of SRF rainbow trout were recorded from 1152 ± 457 to 2897 ± 607 U L^−1^ and were significantly higher than those in normal males (25–120 U L^−1^; Table 4; [54,56]). The high levels of LDH in SRF seminal plasma could be related to LDH absorption by spermatozoa or the storage of this enzyme in the testis during maturation [56].

#### 4.3.3. Oxidative Stress in SRF Semen

Oxidative stress is a key factor in the pathophysiology of semen [93,94]. Oxidative stress arises as a result of the excessive production of reactive oxygen species (ROS), which overcomes the antioxidant defence system and can lead to cellular pathology [95]. Due to this injuring potential, the amount of ROS is precisely controlled by cell-specific antioxidant systems to avoid an oxidative stress as a result of an imbalance in the production and detoxification of ROS [96]. The unique structure of spermatozoa makes them susceptible to oxidative stress, mostly due to the low content of cytoplasm, a rich source of antioxidative enzymes. Oxidative stress leads to several pathological changes, including damage to the plasmatic membranes and DNA.

#### 4.3.4. ROS and Lipid Peroxidation

ROS include highly reactive molecules such as nitric oxide radical (NO^•^), superoxide ion radical (O_2_^•−^), hydroxyl radical (OH^•^), and peroxyl radical (ROO^•^), as well as singlet oxygen (^1^O_2_). These molecules can interact with proteins, lipids, DNA, and RNA, promoting cell injury at several levels. Excessive ROS production leads to the peroxidation of polyunsaturated fatty acids, resulting in the production of aldehydes, such as 4-hydroxynonenal, acrolein, and malondialdehyde [97]. Lipid peroxidation values in the seminal plasma of SRF rainbow trout were found to be within the range of 13.9 ± 5.0–48.5 ± 17.3 × 10^−3^ nmol mg^−1^ protein, significantly lower than those in the seminal plasma of normal males (46.3 ± 12.1–270.0 ± 70.6 × 10^−3^ nmol mg^−1^ protein; [91]). Low levels of lipid peroxidation in SRF seminal plasma can be explained by high levels of total antioxidant capacity (TAC; see below). Consequently, the ROS^+^ values in fresh semen of SRF rainbow trout were found to be 3.3% ± 2.8% and did not differ from those recorded for normal males (3.4% ± 1.2%; [55]). This suggest that under normal physiological conditions, spermatozoa are better protected both in normal and SRF semen.

#### 4.3.5. Total Antioxidant Capacity

The evaluation of antioxidant capacity of seminal plasma is mainly evaluated from the perspective of particular antioxidants. This strategy is time-consuming owing to the necessity of several compounds for analysis and the changeable levels of individual antioxidants [98]. An alternative methodical strategy is to measure the TAC. The TAC in the seminal plasma of SRF rainbow trout was 5.2 ± 1.8 mM Trolox, which is significantly higher than that recorded for normal male rainbow trout (1.8 ± 0.3 mM Trolox; [55]). These results are in accordance with the results of [91]; however, in their study, lower levels of TAC were recorded (0.063–0.147 mM Trolox). It should be noted that the measurement of TAC is possible not only in the seminal plasma but also in the extracts of spermatozoa [55]. In SRF rainbow trout, the TAC in the extracts of spermatozoa was 1.9 ± 0.5 mM Trolox, which was significantly lower than that in normal male rainbow trout (2.8 ± 0.4 mM Trolox; [55]). These results suggest significant differences between SRFs and normal males in terms of antioxidative protection in the seminal plasma and spermatozoa. It is possible that the lower protection in the SRF spermatozoa is compensated for by higher TAC values in the seminal plasma.

#### 4.3.6. Enzymatic Antioxidants

In fish semen, enzymatic antioxidants, such as catalase (CAT), glutathione reductase, methionine reductase, and superoxide dismutase have been identified [98]. Catalase and superoxide dismutase are the first-line antioxidant defence system that plays an important role in the detoxification of ROS to avert oxidative damage [96,98]. The knowledge concerning such antioxidants in SRF semen is very limited; only the CAT values in SRF rainbow trout have been determined (0.2 ± 0.1–0.4 ± 0.2 kU mg^−1^ protein) and were not different from those recorded for normal males (0.4 ± 0.3–0.5 ± 0.3 kU mg^−1^ protein [91]).

#### 4.3.7. DNA Fragmentation

The quality of sperm DNA is an important indicator of the accurate transmission of genetic material from one generation to the next [99]. Oxidative stress is one of the main mechanisms responsible for DNA strand fragmentation [100,101]. The DNA fragmentation of SRF rainbow trout was found to be between 6.1% ± 0.3% and 8.1% ± 2.3% [67,102]. These results seem to be much lower than those obtained for normal male rainbow trout which are within the range of 11.2% ± 9.2–32.0% ± 1.2% [103,104,105]. It is possible that high TAC values in the seminal plasma (see above) can efficiently protect the sperm DNA of SRF from oxidative attack.

## 5. Factors Determining Semen Quality

### 5.1. Introduction

SRF sperm quality parameters are characterised by high variabilities (as indicated above, see Table 3), both between species as well as within one species of SRF. The source of these variabilities should be the different degrees of sex reversion resulting in differences in macroscopic forms [27]. Moreover, other important factors causing variability in semen quality are the seasonality (both within the reproductive season and between seasons), strain, age of fish, and semen collection method [54,57,91,106,107].

### 5.2. Season

The differences in the sperm quality of SRF rainbow trout have been demonstrated during the reproductive season [54,57,91] as well as between the seasons of the year (spring and winter; [106]). The latter study recorded higher sperm motility values for semen samples collected in spring than for those collected in winter [106]. Predictably, the highest sperm motility, VCL, and ALH are recorded in the middle of the reproductive season [54]. In this period, the semen samples are characterised by the lowest values of protein concentration which likely reflect the dilution of the semen during the final steps of semen maturation [54]. The values of APA, osmolality of seminal plasma, TAC, and CAT activity decrease towards the end of spawning season, presumably due to the ageing of semen [54,57,91]. For this period, the highest values of sperm concentration, linearity of movement, LDH, MDA, and seminal plasma pH were also recorded [54,57,91]. The usefulness of these parameters for elucidating the ageing of SRF semen must be confirmed in further studies. Changes in the semen parameters during the reproductive season seem to be consistent for at least two seasons [91].

### 5.3. Strain Effects

The control of the photoperiod enables the year-round maintenance of mature male fishes [108] and is a commonly used practice in salmonid hatcheries. However, it is well known that such manipulations affect sperm quality [109,110]. The high variability in the fresh sperm quality of different lines of SRF rainbow trout were recently observed (Judycka, unpublished). Such variability seems to be connected to different photoperiod regimes used in hatcheries to provide fish year around. Moreover, differences in fresh sperm quality also resulted in significant differences in the usefulness of sperm of different lines SRF rainbow trout for cryopreservation, which varies from about 20% to nearly 60% [107]. This variability makes the implementation of cryopreserved semen of SRF into hatchery practice challenging.

### 5.4. Other Factors

The age of fish is an important factor influencing the sperm quality of SRF [57]. These authors recommended the use of semen collected from 2–3-year-old SRF rainbow trout for reproduction because their semen is characterised by a higher sperm motility than that of 3–4-year-old fish. Due to the necessity of sperm extraction from the SRF testis, semen quality can be highly influenced by the collection method. It has been demonstrated that the semen extraction method for SRF rainbow trout (homogenisation or scalpel) highly affected the sperm motility [106]. In their study, sperm extracted using a scalpel showed a significantly higher motility than did those obtained by homogenisation. Moreover, it is well known that differences in the sperm quality of SRF rainbow trout can occur even within one hatchery between short periods of time [107].

A high variability in semen characteristics is an inherent feature of SRF semen and is determined by several factors. Elucidating the reason for differences in the sperm quality of SRF is important for fish breeders in regard to controlling spawning, as well as improving short- and long-term sperm storage procedures.

## 6. Maturation of Semen

### 6.1. In Vitro Maturation

SRF spermatozoa are often characterised by having a lower potential for motility than normal male spermatozoa, because they have not undergone several processes that take place in the seminal duct, and such sperm has limited potential to fertilise the egg. However, such potential can be acquired through incubating of sperm suspensions in mineral solutions mimicking seminal plasma that contains bicarbonate at high pH levels for 1–2 h [59,111,112,113]. Bicarbonate is a part of the modified Cortland solution (pH 8.0) used by [3] for the dilution of SRF rainbow trout semen. Incubation the milt of SRF rainbow trout in artificial seminal plasma (ASP) containing 130 mM NaCl, 40 mM KCl, 3.3 mM CaCl_2_, 1.5 mM MgCl_2_, and 2.5 mM NaHCO_3_ at pH 9.5 resulted in the significant improvement of sperm motility from 0–5% to 80% after 2 h of incubation [113]. A significant increase in sperm motility from 26% to 66% after a 2 h incubation of SRF rainbow trout semen in ASP containing 100 mM NaCl, 40 mM KCl, 3 mM CaCl_2_, 1.5 mM MgCl_2_, and 50 mM Tris at pH 8.5 was also recorded [59,112]. Moreover, the incubation in ASP caused an increase in the sperm velocity and trajectory parameters (VCL, VSL, ALH, and LIN), whereas the sperm viability was unaffected. The artificial maturation of SRF sperm is prerequisite for the development of the sperm fertilising ability and this procedure has been successfully implemented into routine hatchery practices for SRFs.

### 6.2. Maturation during Equilibration before Freezing

Extenders used for cryopreservation can also be used for the maturation of SRF semen. Several reports concerning cryopreservation of SRF semen clearly show an increase in sperm motility after the dilution of semen with an extender [58,64,66]. In the mentioned studies, the percentage of sperm motility usually increased from about 50% to 80% after 15 min (equilibration) in SRF rainbow trout and brook trout before cryopreservation in a glucose–methanol (GM) extender. However, despite the decrease of percentage of sperm motility after cryopreservation, the post-thaw sperm motility was on the level of 50%, i.e., the initial motility of fresh semen. Moreover, the sperm fertilising ability of cryopreserved semen is high and is similar to fresh semen of normal males. However, it should be considered that in such studies, good-quality samples are usually selected to ensure good cryopreservation success.

The artificial maturation of semen ensures that it is possible to obtain both fresh diluted/equilibrated and cryopreserved semen with the same high fertilising abilities as the fresh semen of normal males. However, the mechanisms of sperm maturation differ between the media used for maturation [61,65]. The latter suggested that compared to ASP, the maturation in the GM extender is incomplete, and some spermatozoa could enter the eggs but are unable to complete fertilisation. Moreover, it seems that there is an interaction of an unknown mechanism between mineral maturation solutions and the GM extender. The dilution of SRF rainbow trout cryopreserved sperm in maturation solutions developed by [112,113] resulted in a decrease in sperm motility from 57% to about 15% [61]. However, this inhibition is reversed over 60 min of storage. This suggests an interaction between the cryoprotectant and maturation solutions during the cryopreservation of SRF semen.

### 6.3. The Mechanism of Maturation

The existing knowledge concerning the mechanism of the sperm maturation of salmonid fishes originates from the comparative studies of semen from the testes and spermatic duct. Seminal plasma from the spermatic duct is characterised by a higher concentration of bicarbonate and higher pH compared to those of testicular plasma, which seems to be critical for sperm motility acquisition in rainbow trout [106]. It is well known that the cAMP-dependent phosphorylation of proteins, including protein kinase PKA-C, is essential for the initiation of sperm motility in salmonid fishes [114,115]. The bicarbonate and high pH present in maturation solutions were demonstrated to elevate the cyclic adenosine monophosphate (cAMP) levels in spermatozoa [112], which is likely produced by soluble adenylyl cyclase [116]. Summing up, the stimulation of adenylyl cyclase by bicarbonate and high pH seems to be an important part of signal transduction leading to sperm maturation.

## 7. Short-Term Semen Storage

In aquaculture, semen storage is important for the conservation of gametes. Semen can be stored at low temperatures for a short period of time, from a few hours to a few days [117,118]. The main aim of short-term storage is the improvement of reproductive procedures and preservation of semen under adequate conditions to maintain its fertilising capacity. Moreover, in the case of SRFs, it ensures the in vitro maturation of semen, which is especially important in SRF semen due to the one-time-only collection of semen (post-mortem). The simplicity of the method, which requires only the dilution of sperm in the mineral media and storage in a thin layer of semen, in the absence of light, at temperatures between 0 and 4 °C and in an oxygen-rich atmosphere, the short-term storage of SRF rainbow trout sperm makes it a common method in hatchery practices.

Media for short-term storage are based mainly on the chemical composition of the seminal plasma and keep sperm immotile to avoid the rapid concomitant depletion of the energy reserves of spermatozoa [119]. It has been found that the semen of SRF rainbow trout diluted in commercial extender (Storfish; IMV, L’Aigle, France) maintains a fertilising ability similar to the fresh semen after six days of storage [68]. In contrast, a decrease in sperm quality parameters, including an increase in DNA fragmentation from 6% to 15% and a decrease in sperm viability from 90% to 45% after five days of storage in Maturfish (IMV, France) was recorded [102]. However, the sperm fertilising ability was not tested in their study. Dilution in sperm maturation media may be also used for both the maturation and storage of sperm for up to 12 h, maintaining 70% of the sperm motility [113]. The short-term storage of semen without a loss of sperm fertilising ability is well incorporated into hatchery practices, allowing for the better economic use of SRF semen by solving the problems of asynchrony between sexually mature males and females.

## 8. Cryopreservation of SRF Sperm

### 8.1. Benefits of Cryopreservation

The cryopreservation of SRF semen provides several potential advantages to commercial aquaculture. Using this method, the best quality semen collected from sacrificed fish can be successfully used for artificial fertilisation, which can facilitate breeding programmes in the hatcheries. Cryopreservation guarantees the accessibility of semen through the year, irrespective of the season and maturity status of fish [120]. Consequently, it is also possible to perform synchronised artificial cross-fertilisations among strains or related species, which have different terms of maturation. Cryopreservation can also be used for maintaining the sperm of valuable strains of SRFs in sperm banks [107,121] and the transportation of semen [122]. The improvement of cryopreservation procedures, particularly standardising the freezing and thawing processes, would therefore facilitate artificial fertilisation and reduce the stock of SRFs maintained at fish farms.

### 8.2. Cryopreservation Procedures

#### 8.2.1. Procedures Based on the Dilution Ratio

Existing knowledge about the cryopreservation of SRF semen is mainly based on the procedures developed for rainbow trout semen (Table 5). Several reports concerning the cryopreservation of semen clearly show that SRF semen can be successfully cryopreserved, preserving its high fertilising ability. The first attempt was conducted by [12] who cryopreserved the semen of SRF Chinook salmon in pellets using 0.3 M glucose and 10% DMSO and obtained fertilisation rates of about 80%. The use of an extender consisting of glucose, DMSO, and egg yolk led to the fertilising ability of the cryopreserved sperm of SRF rainbow trout being about 60% [24,38]. The cryopreservation of SRF rainbow trout semen can be also performed using multicomponent mineral solution of [123] supplemented with only DMSO [106] or DMSO, egg yolk, and Dan Pro S760 [102]. Recently, several studies concerning the cryopreservation of SRF rainbow trout sperm using a simple GM extender were conducted (0.18 M glucose, 9% methanol; [61,63,64,66]. Using this procedure, the post-thaw sperm motility was within the range of about 30–60% and resulted in a fertilising ability of 70–90%. Moreover, [62] recommended that glucose, sucrose, or trehalose can be used interchangeably for the cryopreservation of semen of SRF rainbow trout, with a post-thaw sperm motility of about 40%. It should be considered that despite the development of a wide range of established protocols for the cryopreservation of SRF semen, these procedures do not allow for the easy use of cryopreserved semen under hatchery conditions. Therefore, the standardisation of cryopreservation protocols by establishing an optimal and constant spermatozoa concentration in straws at a constant cryoprotectant concentration is prerequisite for the implementation of cryopreserved semen into hatchery operations. Such an approach was developed a long time ago for the dairy industry, resulting in the impressive development of cattle breeding.

#### 8.2.2. Development of Standardised Procedure

##### Final Sperm Concentration in Straw

The cryopreservation procedures of SRF semen are mostly based on the dilution ratio of semen in the extender (usually 1:3 or 1:9, see Table 5). Spermatozoa concentrations vary among species, stocks, and even within samples from the same fish depending on the times of the collection during the reproductive season. Consequently, the spermatozoa concentration in the straws after dilution with an extender varies between individuals, which could cause differences in the post-thaw sperm motility and fertilisation conditions with respect to the sperm-to-egg ratio. The standardisation of the freezing and thawing procedure is essential to diminish the variability of results and develop commercial applications for fish sperm cryopreservation. The latter can be conducted through the use of sperm cryopreserved using the protocol based on the standardised sperm concentration in the straws; this should facilitate practical fertilisation because there is no need to adjust the sperm concentration of particular semen samples during fertilisation. Recently, a successful attempt was made to standardise the cryopreservation protocol for SRF rainbow trout and brook trout sperm [58]. The highest post-thaw spermatozoa motility was recorded at a concentration 3.0 × 10^9^ spermatozoa mL^−1^. These values were about two to three times higher than those obtained for normal male rainbow trout and brook trout [124,125]. Therefore, the cryopreservation procedure may be more efficient for semen from SRF than for that from normal males. It should be considered that these values are species-specific and there is an optimal range of spermatozoa concentration in the straws; below and above this range, a decreased cryopreserved sperm motility is observed [58,107,125,126].

##### Final Glucose Concentration in the Straws

As previously mentioned, in most cryopreservation protocols published to date, the use of sperm to extender dilution ratios is preferred [120], which clearly cause variability in the final concentrations of extender compounds in extended semen. Recently, it has been found that comparatively small changes in the glucose concentration in the extender significantly affect the post-thaw spermatozoa motility of SRF rainbow trout and brook trout [58]. Final glucose concentrations of 0.15 and 0.19 M were confirmed to produce the highest spermatozoa motilities after the cryopreservation of SRF rainbow trout and brook trout, respectively [58]. Similar to the spermatozoa concentration, the motility of cryopreserved sperm deteriorated below and above the optimal range of the final glucose concentration. It can be supposed that at lower glucose concentrations, the cryoprotectant provided insufficient protection, while higher glucose concentrations may have had an injurious effect on spermatozoa because of the high osmolality [55,125].

#### 8.2.3. Post-Thaw Storage

##### Effects on Post-Thaw Sperm Motility

Prolonged post-thaw storage is important for aquaculture since a large number of straws can be thawed and used for the fertilisation of a high number of eggs. The semen of SRF rainbow trout can be stored for at least 60 min without a decrease in the sperm motility [58,61,63,67]. This post-thaw storage time is comparable to the interval obtained for the semen of normal males [55]. However, the semen of SRF brook trout can be stored without sperm motility loss for up to 360 min [58]. This period is much longer for SRFs than for normal males, the semen of which can last up to 120 min [63]. This suggests that the duration of post-thaw storage of SRF semen is species-specific; this is in accordance with the previous data determined for the semen of normal males [62,63,127]. The prolonged post-thaw storage of semen without losing sperm fertilising ability is important for improving the organisation of hatchery practices and providing an opportunity for prolonged handling time.

##### Effects on Fertilising Ability of Cryopreserved Sperm

Determining the fertilising ability of post-thaw stored sperm is crucial for the implementation of cryopreserved semen into hatchery practices. Recently, the fertilising ability of the cryopreserved semen of SRF rainbow trout was reported to be within the range of 72–90% and did not decrease after 60 min [58] or 120 min [61] of post-thaw storage. The latter also found that these values were similar to those obtained for the fresh semen of normal males. It should be considered that results concerning the fertilising ability of SRF rainbow trout sperm confirmed the quality of post-thaw stored semen established using sperm motility parameters. In contrast, the fertilising ability of cryopreserved semen of SRF brook trout was low (between 33–41%); however, these values did not differ from those of fresh semen [58]. These values were similar to results published following the use of semen from normal male brook trout under hatchery conditions [128]. Moreover, the fertilising ability of SRF sperm brook trout did not change after 60 min of post-thaw storage at a sperm:egg ratio of 500,000:1 [58]. These results of this ratio did not differ much from those of the 300,000:1 ratio recommended for the fresh semen of normal rainbow trout [129]. This indicated that SRF cryopreserved semen can be efficiently integrated into hatchery practices.

#### 8.2.4. Changes in Sperm Quality Parameters during Cryopreservation

In the course of cryopreservation, spermatozoa need to withstand several serious stresses initiated by the freezing/thawing process [130]. The main agents affecting the survival of spermatozoa are sensitivity to cold shock, cooling and freezing rates, extender composition, and osmotic stress (Watson 2000). Therefore, the cryopreservation of sperm causes irreparable damage to cells, resulting in a significant decrease in sperm quality [131,132].

Sperm motility parameters are the most common predictors used for the evaluation of both fresh and cryopreserved semen; the percentage of sperm motility is a fundamental parameter that is used in almost every cryopreservation study. The percentage of motile spermatozoa in SRF semen is affected by cryopreservation with the use of a GM extender; usually, sperm motility increases after equilibration to about 80% and then decreases to the values recorded for fresh semen (50–60%; [61,64,66]). In contrast, when the sperm motility in fresh semen is extremely high (65–80%), equilibration did not increase the sperm motility and a dramatic decrease (to 30–40%) was recorded after cryopreservation [62,63]. A dramatic decrease in the sperm motility of SRF rainbow trout semen after cryopreservation: From 88–100% to 1–6% and from 69–94% to 18–29% for the semen of SRF rainbow trout obtained in spring and winter, respectively, was also recorded [106]. It should be emphasised that the sperm velocity and trajectory parameters are slightly and differentially influenced by cryopreservation [61,64,66]. Moreover, it has been found that some CASA parameters, such as low LIN, high ALH, and VCL, are positively related to the fertilising ability of SRF rainbow trout cryopreserved sperm [66].

The viability of spermatozoa appears to be subjected to similar changes to the sperm motility, with a significant decrease after cryopreservation from about 90% to 60% [55]. It should be noted that viability values are often higher than motility values; for example, the viability of cryopreserved sperm of SRF rainbow trout was about 90% whereas the sperm motility was 75% at the same time [55]. The high sperm viability indicates the high efficiency of the extender used for cryopreservation in the protection of the sperm plasma membrane from cryoinjuries. Moreover, it has been found that the viability seemed to be useful for monitoring the quality of the cryopreserved semen of SRF rainbow trout; significant regressions in sperm viability with sperm fertilising ability, sperm motility, and ALH were found [66].

The damages to spermatozoa during cryopreservation are—to a great extent—caused by oxidative stress, which arises as a result of the increased generation of ROS and/or a reduction in the number of accessible antioxidants [133]. It has been found that cryopreservation causes increased oxidative stress measured as the production of ROS^+^ from 3% to 7% in SRF rainbow trout sperm [55]. It should be noted that the increased production of ROS during cryopreservation can also cause genome lesions, such as DNA fragmentation [102]. These authors recorded a significant increase in DNA fragmentation (from 6% to 14%) after cryopreservation. The analysis of DNA fragmentation appeared to be useful in the development and improvement of cryopreservation protocols, including the addition of antioxidants to extenders, which may decrease the levels of cryoinjuries [134].

The most important parameter for the evaluation of sperm quality in cryopreserved semen is the sperm fertilising ability. Similar to motility, the fertilising ability of cryopreserved SRF sperm is mostly lower than the values obtained using fresh semen [38,106]. In contrast, the cryopreservation of SRF rainbow trout semen using a GM extender resulted in a high fertilising ability (80–90%), similar to values obtained using fresh semen [61,64,66]. The high fertilising ability obtained in the latter studies can be caused by the use of a GM extender for cryopreservation; it is highly efficient at securing a high sperm motility after thawing, which is reflected in the high sperm fertilising ability.

#### 8.2.5. Vitrification

The vitrification of fish sperm has recently gained attention as an alternative to the conventional cryopreservation of salmonid sperm [67,135,136,137]. This process involves the transformation of an aqueous solution into an amorphous solid with a vitreous aspect without ice crystal formation, which is due to a drastic increase in viscosity during rapid freezing [138]. The vitrification of SRF rainbow trout sperm is not a common method, and its effects—with the simultaneous influence of seminal plasma addition—on sperm quality has only been evaluated once [67]. These authors found that a seminal plasma concentration of 50% in Cortland medium [139] with 10% DMSO, 2% BSA, and 0.13 M sucrose was the most effective for the vitrification of sperm and led to 11% DNA fragmentation and 98% plasma membrane integrity, which are similar values to those obtained for fresh semen. However, the mitochondrial membrane integrity and fertilisation ability of vitrified sperm decreased about two-fold compared to those of fresh semen. It should be noted that for vitrification, only small suspension volumes (up to 30 µL) of spermatozoa are frozen. For SRFs, which produce a few millilitres of semen, this technique seems to be ineffective and requires a large amount of time to freeze and then thaw the sperm suspensions needed for artificial fertilisation. Therefore, together with the decreased fertilising ability of vitrified sperm, the currently available vitrification methods are not suitable alternatives to the conventional freezing of SRF semen.

#### 8.2.6. Implementation of Cryopreserved Sperm into Hatchery Practices

##### Benefits of the Use of Cryopreserved Sperm in Hatcheries

The potential benefits of using the cryopreserved sperm of SRF in hatchery practices include the following: (i) Sperm can be preserved and used when the eggs are accessible; (ii) it enables the use of all volumes of accessible sperm; (iii) broodstock maintenance is simplified—out-of-season spawning can be performed only in females and cryopreserved sperm can be used to fertilise the eggs; (iv) it allows the transportation of gametes between distant fish farms and (v) enables the storing genomes of valuable strains of SRF; and (vi) it enables the revitalisation of SRF lines in hatcheries.

##### Creation of Sperm Banks

Gene cryobanking mostly focuses on the storage of cryopreserved sperm of SRFs [140]. Several sperm banks have been established in Europe, the USA, Brazil, Australia, and New Zealand over the last 30 years; however, information concerning the storage of SRF semen is limited. The sperm bank of SRF brook trout and several lines of SRF rainbow trout have been recently established in a commercial hatchery in Poland [107]. It should be underlined that particular SRF lines are characterised by the different usefulnesses of their sperm for cryopreservation. Since salmonid hatcheries base their production on female monosex stocks, the highly efficient cryopreservation of semen from SRFs can be an important factor for the implementation of cryopreserved semen into routine hatchery practices.

##### Fertilisation of Eggs with the Use of Cryopreserved Sperm

The implementation of cryopreserved sperm into the practical conditions of hatcheries is a great challenge. The main problem is scaling up the fertilisation protocols to meet the needs of hatcheries. Recently, it has been demonstrated that it is possible to fertilise up to 1600 rainbow trout eggs using the cryopreserved semen of SRF rainbow trout at a single application without a decrease in the spermatozoa fertilising ability [64]. If we accept that one female rainbow trout (weight of 1.5 kg) produces about 3000 eggs, the fertilisation of that number of eggs at a sperm/egg ratio of 1,000,000:1 can be performed using just two 0.5 mL straws of SRF sperm cryopreserved using a standardised protocol, which ensures that 3 × 10^9^ spermatozoa mL^−1^ is obtained. However, the greatest challenge is the implementation of cryopreserved sperm for mass fertilisation. In hatcheries, the fertilisation of eggs is mainly performed with larger amounts of eggs, e.g., 120,000–140,000. The fertilisation of such amounts of eggs with cryopreserved sperm would require the use of 80 to 93 straws (0.5 mL; see above). Recently, sperm samples of gynogenetic rainbow trout, which were cryopreserved in 2016, were used for the revitalisation of rainbow trout lines after two years of storage [107]. A total of 120,000 eggs (10 L) were fertilised with a fertilisation rate of about 80%, similar to that of the control in which eggs were fertilised with fresh sperm. Moreover, Ciereszko et al. (unpublished) performed the fertilisation of two 14 L portions of rainbow trout eggs (approximately 140,000–168,000 eggs) using cryopreserved sperm of SRF rainbow trout at sperm/egg ratios of 1,000,000:1 and 500,000:1. The obtained results (80%) were similar for both sperm/egg ratios and were close to the values obtained for fresh sperm. This strongly suggests that the cryopreserved semen of SRF could potentially be implemented into breeding programmes based on the crossing of selected SRFs with individual females after confirmation of the rearing performance of larvae. Moreover, the use of sperm cryopreserved using the protocol based on the standardised sperm concentration in the straw should facilitate practical fertilisation because there is no need to adjust the sperm concentration of particular semen samples during fertilisation.

## 9. Proteomics of SRF Sperm

### 9.1. Proteomic Comparison of Normal Male and SRF Testicular Semen of Rainbow Trout

The use of high-throughput proteomic techniques for either the profiling or comparative studies of spermatozoa and seminal plasma proteins have successfully been implemented for various fish species (reviewed by [141]). One of the technologies is gel-based proteomics founded on the separation of proteins by one-dimensional electrophoresis according to their molecular weight, followed by high-performance liquid chromatography electrospray ionisation tandem mass spectrometry (LC-MS/MS), or two-dimensional electrophoresis/two-dimensional difference gel electrophoresis (2D-DIGE) with the application of fluorescent dyes according to their isoelectric point and molecular weight. Two-dimensional difference gel electrophoresis followed by time-of-flight (ToF) Autoflex-ToF/ToF mass spectrometer (MALDI-ToF/ToF) identification was employed to identify differentially abundant proteins in the testicular semen proteome of SRFs and testicular semen proteome of normal male rainbow trout [60]. The analysis of SRF and normal male sperm using 2D-DIGE technology led to the detection of 61 protein spots with a significant difference in their relative abundance when comparing normal male and SRF spermatozoa, 54 of which were identified as different proteins. A total of 28 proteins were found to be overexpressed in the testicular SRF sperm compared to in normal male sperm, whereas 26 proteins were enriched in the testicular male spermatozoa compared to in the SRF sperm. Bioinformatic analysis indicated pathways related to protein remodelling (unfolded protein response) for SRF sperm and pathways associated with energy production (TCA cycle and aspartate degradation) for normal male spermatozoa. It was suggested that normal male and SRF sperm are characterised by different metabolic profiles, and the higher expression levels of proteins involved in energy metabolism (creatine kinase, isocitrate dehydrogenase, malate dehydrogenase) may be the foundation for more efficient energy synthesis in normal male sperm. It is also proposed that the masculinisation process of genetic females leads to inferior spermatogenesis, which does not produce a fully developed system for generating the energy required for sperm movement. The presence of high levels of proteins related to remodelling (T-complex protein, heat shock 70 kDa protein, valosin-containing protein) suggests that spermatogenesis and spermiogenesis have not been completed in SRF and may indicate disturbances to the development of spermatozoa, which leads to the lower quality of SRF semen compared to that of normal males. At the same time, masculinisation seemed to not have a significant impact on the seminal plasma proteome, since no differences were found between the protein profiles of seminal plasma in normal males and SRFs.

### 9.2. Changes in the SRF Rainbow Trout Sperm Proteome after In Vitro Incubation in ASP

Salmonid fish spermatozoa acquire the potential for motility during their transition from the testis to the spermatic duct. During this transition, increases in the concentration of bicarbonate and pH take place, which seem to be essential for sperm motility acquisition in rainbow trout [112]. SRF milt resembles the testicular milt of normal males [3]. Therefore, SRF testicular semen is immature with no or low potential for motility. As indicated above, such potential can be acquired through the artificial in vitro maturation/incubation of testicular sperm suspensions in buffered-saline solutions with high pH, mimicking seminal plasma and containing bicarbonate.

**Figure 4:**
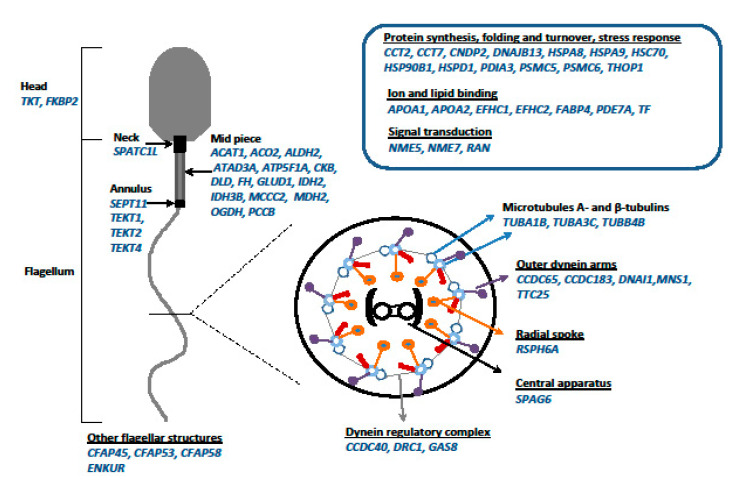
Changes in the sex-reversed female (SRF) rainbow trout proteome after in vitro incubation in artificial seminal plasma (ASP).

The incubation of sperm in ASP resulted in increased sperm motility parameters and had no effect on sperm viability [59]. At the same time, these authors also found profound changes in the sperm protein profile after in vitro maturation (Figure 4). The analysis of fresh and 2-h-incubated sperm in ASP using 2D-DIGE led to the identification of 113 protein spots in SRF sperm, 31 of which were more abundant in fresh sperm, and 85 of which were enriched in SRF sperm. Most of the proteins that change in abundance after sperm incubation in ASP were involved in various pathways and functions, including cytoskeleton and cell movement (CCDC40, coiled-coil domain-containing protein 40; CCDC183, coiled-coil domain-containing protein 183; DRC1, dynein regulatory complex protein 1; CFAP45, cilia- and flagella-associated protein 45; CFAP53, cilia- and flagella-associated protein 53; TEKT2, tektin-2-like isoform X1; TEKT1, tektin-1-like isoform X1; SPATC1L, speriolin-like protein; TUBB4B, tubulin alpha, testis-specific; TTC25, tetratricopeptide repeat protein 25-like; SPAG6, sperm-associated antigen 6; and RSPH6A, radial spoke head protein 6 homolog A-like), metabolism and energy production (OGDH, 2-oxoglutarate dehydrogenase, mitochondrial isoform X3; ACO2, aconitate hydratase, mitochondrial-like; GLUD1, glutamate dehydrogenase, mitochondrial-like; FH, fumarate hydratase, mitochondrial-like; IDH2, isocitrate dehydrogenase 2-1 (NADP+), mitochondrial; ACAT1, acetyl-CoA acetyltransferase, mitochondrial; and CKB, creatine kinase), protein folding, turnover (HSP90B1, 94 kDa glucose-regulated protein precursor; THOP1, thimet oligopeptidase-like; HSPA9, stress-70 protein, mitochondrial-like; HSC70, heat shock cognate 70 kDa protein-like isoform X1; PSMC5, 26S protease regulatory subunit 8 isoform X2; PSMC6, proteasome (prosome, macropain) 26S subunit, ATPase, 6, partial; and CCT7, T-complex protein 1 subunit eta), and molecule binding (EFHC2, EF-hand domain-containing family member C2; APOA 1, apolipoprotein A-I-1 precursor, apolipoprotein A-I-2 precursor). The number of substantial changes in the sperm protein profile associated with cytoskeleton, flagella, and cell movement, as well as metabolic proteins, points out modifications to the sperm motility apparatus during the short artificial in vitro maturation period.

## 10. Concluding Remarks

At present, the majority of salmonid fish aquaculture production is primarily based on the use of SRF semen for the production of all-female populations and female triploids. However, despite the significant progress that has been made in the development of sex reversion procedures, the major challenge is still obtaining high quality semen. The major factors influencing semen quality are the season, age of fish, and method of semen extraction from the testes. Moreover, the different lines maintained in hatcheries are characterised by the high variability in semen quality. Therefore, sperm maturation protocols that significantly improve sperm quality are successfully implemented into hatchery practices. Moreover, the development of efficient semen cryopreservation procedures offers a powerful tool for the breeding of SRFs and providing a constant supply of high-quality cryopreserved semen year-round. However, the implementation of cryopreserved semen into hatchery practices is a significant challenge owing to the limited knowledge of fish breeders regarding the use of cryopreserved semen and lack of personnel skilled in performing high-throughput cryopreservation processes and the fertilisation of eggs using cryopreserved sperm.

The experience gained during recent years has led to the development of procedures resulting in the production of functional SRFs of salmonid fish. This technique could increase the efficiency of hatchery operations by reducing the accidental harvesting of immature males. Moreover, a protocol based on immersion administration to fish (rather than oral administration) would simplify the capture of the steroid before it reaches the environment. To the best of our knowledge, the main obstacle for implementing the production of functional SRFs is the necessity to have 100% females for sex reversal (obtained either by gynogenesis or from all-female populations). It should be considered that recent progress in the selective breeding of rainbow trout greatly improved the marketable size of fish and may offer an alternative to sex control and triploid induction strategies to produce fish (both males and females) that can achieve market weight before sexual maturation [142]. This approach does not require the use of hormonal treatment and sex reversal, and only time will tell whether it will complement the current strategy for SRF production and their use in salmonid culture or completely replace SRF production.

## Figures and Tables

**Table 1:** Examples of the development of the reproductive system in a salmonid sex-reversed female population after hormonal treatment.

Species	Hormone/Method of Application	Functional Male (%)	Dysfunctional Male (%)	Female (%)	Bisexual (%)	Sterile/ Immature (%)	Reference
Rainbow trout *(Oncorhynchus mykiss)*	MT/(6 ppm) feed	5.3	54.4	36.7	1.8	1.8	[23]
	OHA/(20 ppm) feed	1.6	93.4	1.7	1.6	1.7	
	OHA/(20 ppm) feed	3.0	89.6	–	7.4	–	[27]
Brook trout *(Salvelinus fontinalis)*	MT/(400 µg·L^−1^) immersion	17.0	–	–	–	83.0	[28]
	MT/(400 µg·L^−1^) immersion and (3 mg·kg^−1^) feed	31.0	–	–	8.0	61.0	
	MT/(400 µg·L^−1^) immersion and (3 mg·kg^−1^) feed		14.0		1.0	85.0	[6]

MT, 17 α-methyltestosterone, OHA, 17 β-hydroxyandrostenedione, –, not obtained.

**Table 2:** Examples of masculinisation protocols in salmonid fish.

Species	Hormone/Dose	Treatment	Main Outcome	Reference
Arctic charr *(Salvelinus alpinus)*	MDHT/10 mg L^−1^	Weekly immersion 140 °C-days post-hatch	90% males	[5]
	MDHT/0.5 mg kg^−1^	Feeding 140–600 °C-days post-hatch	100% males	
Atlantic salmon *(Salmo salar)*	MT/1 or 3 mg kg^−1^	Feeding 800 °C-days	100% males	[31]
	MDHT/1 mg kg^−1^	Feeding 800 °C-days	100% males	
	MDHT/0.4 mg L^−1^	Immersion 7 and 14 d after hatch	100% males	
Brook trout *(Salvelinus fontinalis)*	MDHT/0.5 mg L^−1^	Immersion 10 d after hatch	up to 45% males	[32]
	MDHT/0.5 mg kg^−1^	Feeding 60 d beginning at first feeding	100% fem. progeny	
	MT/0.4 mg L^−1^	Immersion 4 weekly starting 1 week	100% males	[6]
	MT/3 mg kg^−1^	before hatching and feeding 800 °C-days from the first feeding		
	MT/0.4 mg L^−1^	Immersion on 6th and 4th day pre hatching	75% males	[28]
Brown trout *(Salmo trutta)*	MT/3 mg kg^−1^	Feeding for 800 °C-days	>90% males	[33]
Chinook salmon *(Oncorhynchus tshawytscha)*	MDHT/0.4 mg L^−1^	Immersion on 3 days after 50% hatch	100% males	[20]
	MT/0.4 mg L^−1^	Immersion on 520 °C-days and 620 °C-days after hatching	71% functional males	[34]
Coho salmon *(Oncorhynchus kisutch)*	MT/0.2 mg L^−1^	Two immersions weekly, starting during final hatching	46% functional males	[7]
Rainbow trout *(Oncorhynchus mykiss)*	OHA/20 mg kg^−1^	60 days from the first feeding	96.6% males	[23]
	OHA/0.4 mg L^−1^	Immersion 20 dpf for 2 h and	100% functional males	[24]
	OHA/3 mg kg^−1^	60 days from the first feeding		
	OHA/10 mg kg^−1^	3 months from the first feeding	100% functional males	[35,36]
	MT/3 mg kg^−1^	60 days from the first feeding	87% males	[37]
	MT/0.25 mg kg^−1^	80 days from the first feeding 1024 °C-days	functional males	[38]
	MT/0.4 mg L^−1^	2 h at 1 week post-hatching and weekly for 5 weeks, starting at the onset of first feeding	~50% functional males	[39]

MDHT, 17 α-methyldihydrotestosterone; MT, 17 α-methyltestosterone; OHA, 17 β-hydroxyandrostenedione.

**Table 3:** Characteristics of the fresh semen in salmonid SRF.

Species/ Spawning	Type of Semen	Sperm Concentration (×10^9^ spz mL^−1^) or Spermatocrit (%)	Sperm Motility (%)	VCL (μm s^−1^)	VSL (μm s^−1^)	VAP (μm s^−1^)	ALH (μm)	LIN (%)	Viability (%)	Seminal Plasma Osmolality (mOsm kg^−1^)	Reference
Atlantic salmon *(Salmo salar)*											
Winter	testicular	38.4 ± 1.1	57.2 ± 36.5	155.3 ± 48.6	87.5 ± 31.0	139.4 ± 41.5		63.9 ± 12.8	71.6 ± 7.6		[52]
Brook trout *(Salvelinus fontinalis)*											
November	testicular	29.0 ± 7.0	59.0 ± 8.0	170.0 ± 17.0	20.0 ± 6.0	68.0 ± 6.0	20.0 ± 2.0	13.0 ± 4.0		307 ± 9	[58]
		30.0 ± 4.0	53.0 ± 7.0	155.0 ± 13.0	25.0 ± 7.0	73.0 ± 12.0	20.0 ± 3.0	18.0 ± 5.0		302 ± 4	
Chinook salmon *(Oncorhynchus tshawytscha)*											
October	stripped	0.005	80			110					[44]
Coho salmon *(Oncorhynchus kisutch)*											
Fall	stripped	20%	>30			60					[7]
	testicular	80%	>10			35					
Rainbow trout *(Oncorhynchus mykiss)*											
April	testicular	32.6 ± 2.9	26	~110	~14	~60	~11	~14	~90	287 ± 8	[59]
January	testicular	31.8 ± 3.2	77.3 ± 6.9	227.8 ± 30.2					87.5 ± 1.0	299 ± 8	[55]
November	testicular	28.0 ± 3.0	59.0 ± 17.0	142.0 ± 22.0	40.0 ± 15.0	86.0 ± 21.0	15.0 ± 2.0	27.0 ± 6.0		303 ± 3	[58]
		26.0 ± 4.0	69.0 ± 11.0	123.0 ± 14.0	20.0 ± 5.0	61.0 ± 11.0	16.0 ± 3.0	17.0 ± 2.0		304 ± 4	
		31.0 ± 9.0	56.0 ± 10.0	135.0 ± 31.0	17.0 ± 6.0	51.0 ± 13.0	16.0 ± 4.0	13.0 ± 4.0		305 ± 4	
First spawning	testicular										[57]
December		22.77 ± 1.05								335 ± 8	
January		28.11 ± 1.5									
February		30.44 ± 1.75								310 ± 3	
Second spawning											
December		25.15 ± 3.01								342 ± 8	
January		36.83 ± 2.94									
February		37.09 ± 3.30								311 ± 4	
April	testicular	44.3 ± 7.0	36.2 ± 16.1	112.5 ± 19.5	28.7 ± 11.8	63.1 ± 7.0	10.5 ± 3.3	24.7 ± 8.9	95.7 ± 2.7	305 ± 12	[60]
April	testicular	31.22 ± 7.55	52.8 ± 9.4	173.3 ± 25.4	46.5 ± 11.6	97.4 ± 18.9	21.4 ± 4.1	25.8 ± 6.0			[61]
December	testicular	32.2 ± 7.0	75	140	25	70	19	19		314 ± 12	[62]
December	testicular	31.6 ± 6.2	65	150	45	80	16	25		283 ± 37	[63]
December	testicular	32.7 ± 3.4									[64]
May	testicular	30.1 ± 5.0	49.4 ± 7.2	140	22	60	17	16		290 ± 7	[65]
December	testicular	42.8 ± 4.2	50	135	30	70	16	21		300 ± 8	[66]
	testicular	26.4 ± 2.4	64.0 ± 10.0								[47]
		35.1 ± 6.5	71.0 ± 11.0								
	testicular	12.0 ± 1.4	>80%						99.1 ± 5.1		[67]
February motility:	testicular										[54]
<25%		37.5 ± 2.5	17.7 ± 6.5	130.0 ± 19.3	23.6 ± 7.6		16.9 ± 3.6	21.3 ± 7.7	~90.0	326.3 ± 10.9	
25–50%		36.6 ± 6.2	41.6 ± 6.4	122.5 ± 14.3	24.9 ± 6.6		15.3 ± 3.0	23.3 ± 6.6	~90.0	323.5 ± 11.8	
>50%		32.5 ± 5.0	64.7 ± 11.9	149.2 ± 15.8	31.5 ± 8.2		8.9 ± 3.0	21.9 ± 5.1	~90.0	320.6 ± 8.4	
February-beginning	testicular	35.5 ± 5.7	41.1 ± 20.2	133.2 ± 20.1	26.5 ± 8.2		17.0 ± 3.5	22.3 ± 6.9		323.6 ± 10.9	[56]
April-middle		32.9 ± 5.5	59.5 ± 18.1	151.4 ± 18.1	30.9 ± 12.9		19.1 ± 2.5	20.7 ± 6.6		316.4 ± 15.9	
April-end of the spawning season		38.8 ± 9.4	37.2 ± 22.2	114.6 ± 23.1	28.0 ± 8.7		15.5 ± 4.7	28.3 ± 9.5		308.9 ± 9.9	
	testicular	21.6 ± 2.7	73.1	127.5	34.6	74.1	15.0	26.9		308 ± 20	[49]
	testicular		13.6 ± 19.3								[68]
December and January	testicular	22.5 ± 8.3									[3]

VCL, curvilinear velocity; VSL, straight-line velocity; VAP, average path velocity; ALH, amplitude of lateral head displacement; LIN, linearity.

**Table 4:** Biochemical characteristics of the SRF rainbow trout (*Oncorhynchus mykiss*).

Spawning	Protein Concentration (mg mL^−1^)	Antitrypsin Activity (U L^−1^)	Lactate Dehydrogenase Activity (U L^−1^)	Reference
First spawning				[57]
December	7.8 ± 1.9			
January	6.1 ± 2.0			
February	4.0 ± 1.6			
Second spawning				
December	8.2 ± 1.3			
January	5.7 ± 1.4			
February	3.4 ± 1.4			
April	27.3 ± 3.0			[60]
February				[54]
Motility:				
<25%	5.4 ± 1.9	1453 ± 496	1709 ± 658	
25–50%	4.5 ± 1.4	1235 ± 544	1479 ± 632	
>50%	3.5 ± 1.3	849 ± 210	1152 ± 457	
February-beginning	4.3 ± 1.8	1117 ± 437	1408 ± 636	[56]
April-middle	2.9 ± 1.1	794 ± 270	1169 ± 513	
April-end of the spawning season	3.6 ± 1.9	748 ± 416	2897 ± 607	
	7.7 ± 5.1			[49]

**Table 5:** Cryopreservation protocols for SRF semen.

Procedure Based on the Dilution Ratio							
SRF Species	Extender Composition	Cryopreservation Procedure		Post-Thaw Motility (%)	Post-Thaw Viability (%)	Fertilising Ability (%)	References
		Dilution Ratio	Straw Size				
Rainbow trout (*Oncorhynchus mykiss*)	5.4% glucose, 9% DMSO, 10% egg yolk	1:3	1.7 mL microfuge tube			0–62% of control	[24]
	7% DMSO, 10% egg yolk, 7.5 mg mL^−1^ Dan Pro S760 in the mineral solution #6 from Erdahl and Graham (1980; 0.7 mM CaCl_2_ × 2H_2_O, 1.08 mM MgCl_2_ × 6H_2_O, 1.49 mM Na_2_HPO_4_, 34.3 mM KCl, 100 mM NaCl, 0.52 mM citric acid, 55.5 mM glucose, 20 mL KOH solution 226 mM, 20 mL bicine solution 324 mM, 323 mOsm kg^−1^, pH 7.4)	1:3	0.5 mL	spring strain 1–6%		72–78	[106]
				winter strain 18–29%		74–84	
	80% of 5.4% glucose, 10% egg yolk, 10% DMSO	1:3	0.5 mL			56 ± 18	[38]
	#6 Erdahl and Graham (1980; see above) and 7% DMSO	1:3	0.5 mL		69 ± 4		[102]
	0.18 M glucose, 9% methanol	1:9	0.25 mL	55	56	80	[66]
				57 ± 7		91 ± 2–94 ± 2	[64]
				~30			[63]
				57 ± 9		72–87	[61]
	0.18 M glucose, 9% methanol	1:9	0.25 mL	~40			[62]
	0.18 M sucrose, 9% methanol			~40			
	0.18 M trehalose, 9% methanol			~40			
Chinook salmon (*Oncorhynchus tshawytscha*)	0.3 M glucose, 10% DMSO	1:3	pellets			76 ± 8	[18]
**Standardized Procedure**							
**SRF Species**	**Final Concentration of Cryoprotectants**	**Final Spermatozoa Concentration in Straw (×10^9^ mL^−1^)**	**Straw Size**	**Post-Thaw Motility (%)**	**Post-Thaw Viability (%)**	**Fertilising Ability (%)**	**References**
Rainbow trout (*Oncorhynchus mykiss*)	0.15 M glucose, 7.5% methanol	3	0.5 mL	49		81–90	[58]
				~60	~70		[55]
Brook trout (*Salvelinus fontinalis*)	0.19 M glucose, 7.5% methanol	3	0.5 mL	55		33–41	[58]

## Data Availability

No new data were created or analysed in this study. Data sharing is not applicable to this article.

## Abbreviations

ACAT1	acetyl-CoA acetyltransferase, mitochondrial
ACO2	aconitate hydratase, mitochondrial-like;
ALDH2	aldehyde dehydrogenase, mitochondrial;
APOA1	apolipoprotein A-I-1 precursor, apolipoprotein A-I-2 precursor;
APOA2	apolipoprotein A-II precursor;
ATAD3A	ATPase family AAA domain-containing protein 3A;
ATP5F1A	ATP synthase subunit alpha, mitochondrial-like;
CCDC183	coiled-coil domain-containing protein 183;
CCDC40	coiled-coil domain-containing protein 40;
CCDC65	coiled-coil domain-containing protein 65;
CCT2	T-complex protein 1 subunit beta;
CCT7	T-complex protein 1 subunit eta;
CFAP45	cilia- and flagella-associated protein 45;
CFAP53	cilia- and flagella-associated protein 53;
CFAP58	cilia- and flagella-associated protein 58;
CKB	creatine kinase;
CNDP2	cytosolic non-specific dipeptidase-like isoform X2;
DLD	dihydrolipoyl dehydrogenase, mitochondrial-like;
DNAI1	dynein intermediate chain 1;
DNAJB13	dnaJ homolog subfamily B member 13;
DRC1	dynein regulatory complex protein 1;
EFHC1	EF-hand domain-containing protein 1;
EFHC2	EF-hand domain-containing family member C2;
ENKUR	enkurin isoform X1;
FH	fumarate hydratase, mitochondrial-like;
FKBP2	peptidyl-prolyl cis-trans isomerase FKBP2;
GAS8	growth arrest-specific protein 8;
GLUD1	glutamate dehydrogenase, mitochondrial-like;
HSC70	heat shock cognate 70 kDa protein-like isoform X1;
HSP90B1	94 kDa glucose-regulated protein precursor;
HSPA8	heat shock cognate 70 kDa protein-like;
HSPA9	stress-70 protein, mitochondrial-like;
HSPD1	60 kDa heat shock protein, mitochondrial precursor;
IDH2	isocitrate dehydrogenase 2-1 (NADP+), mitochondrial;
IDH3B	isocitrate dehydrogenase [NAD] subunit beta, X2;
MCCC2	methylcrotonoyl-CoA carboxylase beta chain, mitochondrial, partial;
MDH2	malate dehydrogenase 2-1, NAD (mitochondrial);
MNS1	meiosis-specific nuclear structural protein 1-like;
NME5	nucleoside diphosphate kinase homolog 5 isoform X3;
NME7	nucleoside diphosphate kinase 7;
OGDH	2-oxoglutarate dehydrogenase, mitochondrial isoform X3;
PCCB	propionyl-CoA carboxylase beta chain, mitochondrial-like;
PDE7A	high affinity cAMP-specific 3’,5’-cyclic phosphodiesterase 7A isoform X2;
PDIA3	disulfide-isomerase A3 precursor;
PSMC5	26S protease regulatory subunit 8 isoform X2;
PSMC6	proteasome (prosome, macropain) 26S subunit, ATPase, 6, partial;
RAN	GTP-binding nuclear protein Ran;
RSPH6A	radial spoke head protein 6 homolog A-like;
SEPT11	septin-11-like isoform X2;
SPAG6	sperm-associated antigen 6;
SPATC1L	speriolin-like protein;
TEKT1	tektin-1-like isoform X1;
TEKT2	tektin-2-like isoform X1;
TEKT4	tektin-4-like;
THOP1	thimet oligopeptidase-like;
TKT	transketolase-like;
TTC25	tetratricopeptide repeat protein 25-like;
TUBA1B	tubulin alpha chain;
TUBA3C	tubulin alpha chain, testis-specific;
TUBB4B	tubulin alpha, testis-specific.
NME5	nucleoside diphosphate kinase homolog 5 isoform X3;
NME7	nucleoside diphosphate kinase 7;

## References

[1] Donaldson E.M. (1996). Manipulation of reproduction in farmed fish. Anim. Reprod. Sci..

[2] Pandian T., Sheela S. (1995). Hormonal induction of sex reversal in fish. Aquaculture.

[3] Geffen A.J., Evans J. (2000). Sperm traits and fertilization success of male and sex-reversed female rainbow trout (*Oncorhynchus mykiss*). Aquaculture.

[4] Baroiller J.-F., D’Cotta H. (2016). The reversible sex of gonochoristic fish: Insights and consequences. Sex. Dev..

[5] Chiasson M., Benfey T.J. (2007). Gonadal differentiation and hormonal sex reversal in arctic charr (Salvelinus alpinus). J. Exp. Zool. Part A Ecol. Genet. Physiol..

[6] Haffray P., Petit V., Guiguen Y., Quillet E., Rault P., Fostier A. (2009). Successful production of monosex female brook trout Salvelinus fontinalis using gynogenetic sex reversed males by a combination of methyltestosterone immersion and oral treatments. Aquaculture.

[7] Fitzpatrick J.L., Henry J.C., Liley N.R., Devlin R.H. (2005). Sperm characteristics and fertilization success of masculinized coho salmon (*Oncorhynchus kisutch*). Aquaculture.

[8] Budd A.M., Banh Q.Q.T., Domingos J.A., Jerry D.R. (2015). Sex control in fish: Approaches, challenges and opportunities for aquaculture. J. Mar. Sci. Eng..

[9] Migaud H., Bell G., Cabrita E., McAndrew B., Davie A., Bobe J., Herráez M., Carrillo M. (2013). Gamete quality and broodstock management in temperate fish. Rev. Aquac..

[10] Weber G.M., Lee C.-S., Lamb G.C., DiLorenzo N. (2014). Current and future assisted reproductive technologies for fish species. Current and Future Reproductive Technologies and World Food Production.

[11] Donaldson E.M., Hunter G.A. (1982). Sex control in fish with particular reference to salmonids. Can. J. Fish. Aquat. Sci..

[12] Hunter G.A., Donaldson E.M. (1983). 5 Hormonal sex control and its application to fish culture. Fish Physiol..

[13] Piferrer F. (2001). Endocrine sex control strategies for the feminization of teleost fish. Aquaculture.

[14] Francis R.C. (1992). Sexual lability in teleosts: Developmental factors. Q. Rev. Biol..

[15] Padoa E. (1937). Differenziazione e inversione sessuale (femminilizzazione) di avannotti di trota (*Salmo irideus*) trattati con ormone fol-licolare. Monit. Zool. Ital..

[16] Ashby K.R. (1957). The effect of steroid hormones on the brown trout (*Salmo trutta* L.) during the period of gonadal differentiation. J. Embryol. Exp. Morphol..

[17] Hunter G.A., Donaldson E.M., Goetz F.W., Edgell P.R. (1982). Production of all-female and sterile coho salmon, and experimental evidence for male heterogamety. Trans. Am. Fish. Soc..

[18] Hunter G.A., Donaldson E.M., Stoss J., Baker I. (1983). Production of monosex female groups of chinook salmon (*Oncorhynchus tshawytscha*) by the fertilization of normal ova with sperm from sex-reversed females. Aquaculture.

[19] Devlin R.H., Nagahama Y. (2002). Sex determination and sex differentiation in fish: An overview of genetic, physiological, and environmental influences. Aquaculture.

[20] Piferrer F., Baker I.J., Donaldson E.M. (1993). Effects of natural, synthetic, aromatizable, and nonaromatizable androgens in inducing male sex differentiation in genotypic female chinook salmon (*Oncorhynchus tshawytscha*). Gen. Comp. Endocrinol..

[21] Guiguen Y., Bertho S., Herpin A., Fostier A., Wang H., Piferrer F., Chen S., Chen S.-L., Shen Z.-G. (2019). Sex determination and sex control in salmonidae. Sex Control in Aquaculture.

[22] Demska-Zakęś K., Hliwa P., Matyjewicz P., Zakęś Z. (1999). Effect of 17 alpha-methyltestosterone and 11 beta-hydroxyandrostenedione on the development of reproductive system in rainbow trout (*Oncorhynchus mykiss* Walbaum). Arch. Polish Fish..

[23] Kuźmiński H., Dobosz S. (2010). Effect of sex reversal in rainbow trout (*Oncorhynchus mykiss* Walbaum) using 17α-methyltestosterone and 11β-hydroxyandrostenedione. Arch. Pol. Fish..

[24] Feist G., Yeoh C.-G., Fitzpatrick M.S., Schreck C.B. (1995). The production of functional sex-reversed male rainbow trout with 17α-methyltestosterone and 11 β-hydroxyandrostenedione. Aquaculture.

[25] Feist G., Schreck C.B., Gharrett A.J. (1996). Controlling the Sex of Salmonids.

[26] Yossa R., Bardon-Albaret A., Chiasson M.A., Liu Q., Duston J., Manning T., Benfey T.J. (2019). Controlling preharvest maturity in farmed Arctic char: A review from the Canadian perspective. J. World Aquac. Soc..

[27] Hliwa P., Kuźmiński H., Dobosz S., Nynca J., Dietrich G.J., Ziomek E., Ciereszko A., Zakęś Z., Demska-Zakęś K., Kowalska A. (2011). Gonad morphology and semen quality of rainbow trout (*Oncorhynchus mykiss*) neo-males. New Species in Aquaculture—Reproduction, Breeding, Prophylaxis.

[28] Fatima S., Adams M., Wilkinson R. (2016). Sex reversal of brook trout (*Salvelinus fontinalis*) by 17α-methyltestosterone exposure: A serial experimental approach to determine optimal timing and delivery regimes. Anim. Reprod. Sci..

[29] Fatima S., Adams M.B., Wilkinson R. (2011). Histological study of gonadal development and sex differentiation in Salvelinus fontinalis under Tasmanian climate conditions. Aust. J. Zool..

[30] Valdivia K., Jouanno E., Volff J.-N., Galiana-Arnoux D., Guyomard R., Helary L., Mourot B., Fostier A., Quillet E., Guiguen Y. (2014). High temperature increases the masculinization rate of the all-female (xx) rainbow trout “mal” population. PLoS ONE.

[31] Lee P., King H., Pankhurst N. (2004). Preliminary Assessment of Sex Inversion of Farmed Atlantic Salmon by Dietary and Immersion Androgen Treatments. N. Am. J. Aquac..

[32] Galbreath P.F., Adams N.D., Sherrill L.W. (2003). Successful sex reversal of brook trout with 17α-methyldihydrotestosterone treatments. N. Am. J. Aquac..

[33] Chevassus B., Krieg F. (1992). Effect of the concentration and duration of methyltestosterone treatment on masculinization rate in the brown trout (*Salmo trutta*). Aquat. Living Resour..

[34] Heath D.D., Rankin L., Bryden C.A., Heath J.W., Shrimpton J.M. (2002). Heritability and Y-chromosome influence in the jack male life history of chinook salmon (*Oncorhynchus tshawytscha*). Heredity.

[35] Baron D., Montfort J., Houlgatte R., Fostier A., Guiguen Y. (2007). Androgen-induced masculinization in rainbow trout results in a marked dysregulation of early gonadal gene expression profiles. BMC Genom..

[36] Baron D., Houlgatte R., Fostier A., Guiguen Y. (2008). Expression profiling of candidate genes during ovary-to-testis trans-differentiation in rainbow trout masculinized by androgens. Gen. Comp. Endocrinol..

[37] Atar H.H., Bekcan S., Dogankaya L. (2009). Effects of different hormones on sex reversal of rainbow trout (Oncorhynchus mykissWalbaum) and production of all-female populations. Biotechnol. Biotechnol. Equip..

[38] Ninhaus-Silveira A., Foresti F., Tabata Y.A., Rigolino M.G., Veríssimo-Silveira R. (2006). Cryopreservation of semen from functional sex-reversed genotypic females of the rainbow trout, Oncorhynchus mykiss. Braz. Arch. Biol. Technol..

[39] Weber G.M., Leeds T.D., Schneider R.P. (2020). Sex reversal of female rainbow trout by immersion in 17α-methyltestosterone. Aquaculture.

[40] Barry T.P., Marwah A., Marwah P. (2007). Stability of 17α-methyltestosterone in fish feed. Aquaculture.

[41] Piferrer F., Donaldson E.M. (1992). The comparative effectiveness of the natural and a synthetic estrogen for the direct feminization of chinook salmon (*Oncorhynchus tshawytscha*). Aquaculture.

[42] Johnstone R., MacLachlan P. (1994). Further observations on the sex inversion of Atlantic salmon, Salmo salar L., Using 17α methyl testosterone. Aquac. Res..

[43] Devaux A., Bony S., Plenet S., Sagnes P., Segura S., Suaire R., Novak M., Gilles A., Olivier J.-M. (2015). Field evidence of reproduction impairment through sperm DNA damage in the fish nase (*Chondrostoma nasus*) in anthropized hydrosystems. Aquat. Toxicol..

[44] Lehnert S.J., Heath D.D., Pitcher T.E. (2012). Sperm trait differences between wild and farmed Chinook salmon (*Oncorhynchus tshawytscha*). Aquaculture.

[45] Cousin-Gerber M., Burger G., Boisseau C., Chevassus B. (1989). Effect of methyltestosterone on sex differentiation and gonad morphogenesis in rainbow trout Oncorhynchus mykiss. Aquat. Living Resour..

[46] Baynes S. (1999). Fertilisation procedures for use in all-female brood production. Trout News.

[47] Hliwa P., Bah M.M., Kuźmiński H., Dobosz S., Ciereszko A. (2013). Ultrasound evaluation of the gonadal structure in sex-reversed rainbow trout females. Aquac. Int..

[48] Komen H., Thorgaard G.H. (2007). Androgenesis, gynogenesis and the production of clones in fishes: A review. Aquaculture.

[49] Kowalski R.K., Sarosiek B., Demianowicz W., Judek J., Goryczko K., Dobosz S., Kuźmiński H., Demska-Zakeś K., Babiak I., Glogowski J. (2011). Quantitative characteristics of rainbow trout, Oncorhynchus mykiss, neo-males (XX genotype) and super-males (YY genotype) sperm. World Acad. Sci. Eng. Technol..

[50] Bye V.J., Lincoln R.F. (1986). Commercial methods for the control of sexual maturation in rainbow trout (*Salmo gairdneri* R.). Aquaculture.

[51] Galas J.F., Hejmej A., Glogowski J., Bilińska B. (2009). Morphological and functional alterations in testes and efferent ducts of homogametic rainbow trout Oncorhynchus mykiss walbaum. Ann. N. Y. Acad. Sci..

[52] de Castro P.L., Patil J.G. (2019). Comparative gonad histology and semen quality of normal (XY) and neo-males (XX) of Atlantic salmon (Salmo salar). Aquac. Res..

[53] Petersen C., Söder O. (2006). The sertoli cell—a hormonal target and ‘super’ nurse for germ cells that determines testicular size. Horm. Res. Paediatr..

[54] Nynca J., Kuźmiński H., Dietrich G., Hliwa P., Dobosz S., Liszewska E., Karol H., Ciereszko A. (2012). Changes in sperm parameters of sex-reversed female rainbow trout during spawning season in relation to sperm parameters of normal males. Theriogenology.

[55] Judycka S., Słowińska M., Nynca J., Liszewska E., Dobosz S., Ciereszko A. (2020). Oxidative stress in cryopreserved semen of sex-reversed female and normal male rainbow trout. Aquaculture.

[56] Nynca J., Kuźmiński H., Dietrich G.J., Hliwa P., Dobosz S., Liszewska E., Karol H., Ciereszko A. (2012). Biochemical and physiological characteristics of semen of sex-reversed female rainbow trout (*Oncorhynchus mykiss*, Walbaum). Theriogenology.

[57] Inanan B.E., Yılmaz F. (2018). Seasonal and age-related changes in semen characteristics and composition of seminal plasma in sex-reverse female rainbow trout (*Oncorhynchus mykiss*) in comparison with normal males. Anim. Reprod. Sci..

[58] Judycka S., Nynca J., Liszewska E., Mostek A., Ciereszko A. (2019). Comparative analysis of sperm freezability of sex-reversed female brook trout and sex-reversed female rainbow trout semen. Aquaculture.

[59] Nynca J., Słowińska M., Judycka S., Dobosz S., Ciereszko A. (2020). Acquiring the potential for motility is accompanied by profound changes in the testicular sperm proteome of sex-reversed female and normal male rainbow trout. Aquaculture.

[60] Nynca J., Adamek M., Ciereszko A. (2017). Identification of differentially expressed proteins in testicular semen of sex-reversed female (XX) and normal male (XY) rainbow trout^1^. J. Anim. Sci..

[61] Judycka S., Ciereszko A., Dobosz S., Zalewski T., Dietrich G.J. (2017). Effect of dilution in sperm maturation media and time of storage on sperm motility and fertilizing capacity of cryopreserved semen of sex-reversed female rainbow trout. Gen. Comp. Endocrinol..

[62] Nynca J., Judycka S., Liszewska E., Dobosz S., Grudniewska J., Arai K., Fujimoto T., Ciereszko A. (2016). Utility of different sugar extenders for cryopreservation and post-thaw storage of sperm from Salmonidae species. Aquaculture.

[63] Judycka S., Nynca J., Liszewska E., Dobosz S., Zalewski T., Ciereszko A. (2016). Potassium ions in extender differentially influence the post-thaw sperm motility of salmonid fish. Cryobiology.

[64] Ciereszko A., Dietrich G., Nynca J., Krom J., Dobosz S. (2015). Semen from sex-reversed rainbow trout of spring strain can be successfully cryopreserved and used for fertilization of elevated number of eggs. Aquaculture.

[65] Ciereszko A., Dietrich G.J., Nynca J., Dobosz S., Krom J. (2015). Maturation of spermatozoa from rainbow trout (*Oncorhynchus mykiss*) sex-reversed females using artificial seminal plasma or glucose–methanol extender. Theriogenology.

[66] Dietrich G., Nynca J., Dobosz S., Zalewski T., Ciereszko A. (2014). Application of glucose–methanol extender to cryopreservation of semen of sex-reversed females rainbow trout results in high post-thaw sperm motility and fertilizing ability. Aquaculture.

[67] Figueroa E., Risopatrón J., Sánchez R., Isachenko V., Merino O., Isachenko V., Valdebenito I. (2013). Spermatozoa vitrification of sex-reversed rainbow trout (*Oncorhynchus mykiss*): Effect of seminal plasma on physiological parameters. Aquaculture.

[68] Haffray P., Sambroni E., Enright W.J., Driancourt M.A., Mikolajczyk T., Rault P., Breton B. (2008). Efficiency of GonazonTM in rainbow trout, the first officially approved inducer of ovulation in the EU. Cybium.

[69] Dong Q., Huang C., Tiersch T.R. (2007). Control of sperm concentration is necessary for standardization of sperm cryopreservation in aquatic species: Evidence from sperm agglutination in oysters. Cryobiology..

[70] Rurangwa E., Kime D., Ollevier F., Nash J. (2004). The measurement of sperm motility and factors affecting sperm quality in cultured fish. Aquaculture.

[71] Gallego V., Asturiano J.F. (2019). Fish sperm motility assessment as a tool for aquaculture research: A historical approach. Rev. Aquac..

[72] Boryshpolets S., Kowalski R.K., Dietrich G., Dzyuba B., Ciereszko A. (2013). Different computer-assisted sperm analysis (CASA) systems highly influence sperm motility parameters. Theriogenology.

[73] Hossain M.S., Johannisson A., Wallgren M., Nagy S., Siqueira A.P., Rodriguez-Martinez H. (2011). Flow cytometry for the assessment of animal sperm integrity and functionality: State of the art. Asian J. Androl..

[74] Jamieson B.G.M. (2006). Avian spermatozoa: Structure and phylogeny. Reproductive Biology and Phylogeny of Birds, Part A: Phy-logeny, Morphology, Hormones and Fertilization.

[75] Agnihotri S.K., Agrawal A.K., Hakim B.A., Vishwakarma A.L., Narender T., Sachan R., Sachdev M. (2016). Mitochondrial membrane potential (MMP) regulates sperm motility. In Vitro Cell. Dev. Biol. Anim..

[76] Ogier De Baulny B., Le Vern Y., Kerboeuf D., Maisse G. (1997). Flow cytometric evaluation of mitochondrial activity and membrane integrity in fresh and cryopreserved rainbow trout (*Oncorhynchus mykiss*) spermatozoa. Cryobiology.

[77] Trigo P., Merino O., Figueroa E., Valdebenito I., Sánchez R., Risopatrón J. (2015). Effect of short-term semen storage in salmon (*Oncorhynchus mykiss*) on sperm functional parameters evaluated by flow cytometry. Andrologia.

[78] Auger J., Ronot X., Dadoune J.P. (1989). Human sperm mitochondrial function related to motility: A flow and image cytometric assessment. J. Androl..

[79] Folgerø T., Bertheussen K., Lindal S., Torbergsen T., Øian P. (1993). Andrology: Mitochondrial disease and reduced sperm motility. Hum. Reprod..

[80] Ciereszko A., Dabrowski K. (2000). Effect of ascorbic acid supplement in vitro on rainbow trout sperm viability. Aquac. Int..

[81] Morisawa M., Suzuki K., Morisawa S. (1983). Effect of potassium and osmolarity on spermatozoa motility of salmonid fishes. J. Exp. Biol..

[82] Marshall W.S., Bryson S.E., Idler D.R. (1989). Gonadotropin stimulation of K+ secretion and Na+ absorption by brook trout (*Salvelinus fontinalis*) sperm duct epithelium. Gen. Comp. Endocrinol..

[83] Lahnsteiner F., Patzner R.A., Welsmann T. (1993). The spermatic ducts of salmonid fishes (Salmonidae, Teleostei). Morphology, histochemistry and composition of the secretion. J. Fish Biol..

[84] Schulz R.W., Miura T. (2002). Spermatogenesis and its endocrine regulation. Fish Physiol. Biochem..

[85] Billard R. (1986). Spermatogenesis and spermatology of some teleost fish species. Reprod. Nutr. Dev..

[86] Glogowski J., Kwasnik M., Piros B., Dabrowski K., Goryczko K., Dobosz S., Kuzminski H., Ciereszko A. (2000). Characterization of rainbow trout milt collected with a catheter: Semen parameters and cryopreservation success. Aquac. Res..

[87] Król J., Żarski D., Bernáth G., Palińska-Żarska K., Krejszeff S., Długoński A., Horvath A. (2018). Effect of urine contamination on semen quality variables in Eurasian perch *Perca fluviatilis* L.. Anim. Reprod. Sci..

[88] Dabrowski K., Ciereszko A. (1994). Proteinase inhibitor(s) in seminal plasma of teleost fish. J. Fish Biol..

[89] Ciereszko A., Piros B., Dabrowski K., Kucharczyk D., Łuczyński M.J., Dobosz S., Glogowski J. (1998). Serine proteinase inhibitors of seminal plasma of teleost fish: Distribution of activity, electrophoretic profiles and relation to proteinase inhibitors of blood. J. Fish Biol..

[90] Wojtczak M., Całka J., Glogowski J., Ciereszko A. (2007). Isolation and characterization of α1-proteinase inhibitor from common carp (*Cyprinus carpio*) seminal plasma. Comp. Biochem. Physiol. Part B Biochem. Mol. Biol..

[91] Inanan B., Öğretmen F., Inanan T., Yılmaz F. (2016). Total antioxidant capacity, catalase activity, and lipid peroxidation changes in seminal plasma of sex-reversed female and male rainbow trout (*Oncorhynchus mykiss*) during spawning season. Theriogenology.

[92] Ciereszko A., Dabrowski K. (1994). Relationship between biochemical constituents of fish semen and fertility: The effect of short-term storage. Fish Physiol. Biochem..

[93] Aitken R.J., Krausz C. (2001). Oxidative stress, DNA damage and the Y chromosome. Reproduction.

[94] Bennetts L.E., Aitken R.J. (2005). A comparative study of oxidative DNA damage in mammalian spermatozoa. Mol. Reprod. Dev..

[95] Agarwal A., Prabakaran S.A., Said T.M. (2005). Prevention of oxidative stress injury to sperm. J. Androl..

[96] Cabrita E., Martínez-Páramo S., Gavaia P.J., Riesco M.F., Valcarce D.G., Sarasquete C., Herráez M.P., Robles V. (2014). Factors enhancing fish sperm quality and emerging tools for sperm analysis. Aquaculture.

[97] Pini T., Leahy T., De Graaf S.P. (2018). Sublethal sperm freezing damage: Manifestations and solutions. Theriogenology.

[98] Lahnsteiner F., Mansour N. (2010). A comparative study on antioxidant systems in semen of species of the Percidae, Salmonidae, Cyprinidae, and Lotidae for improving semen storage techniques. Aquaculture.

[99] Twigg J.P., Irvine D.S., Aitken R.J. (1998). Oxidative damage to DNA in human spermatozoa does not preclude pronucleus formation at intracytoplasmic sperm injection. Hum. Reprod..

[100] Labbe C., Martoriati A., Devaux A., Maisse G. (2001). Effect of sperm cryopreservation on sperm DNA stability and progeny development in rainbow trout. Mol. Reprod. Dev..

[101] Aitken R.J., Jones K.T., Robertson S.A. (2012). Reactive oxygen species and sperm function—in sickness and in health. J. Androl..

[102] Pérez-Cerezales S., Martínez-Páramo S., Cabrita E., Martínez-Pastor F., De Paz P., Herráez M. (2009). Evaluation of oxidative DNA damage promoted by storage in sperm from sex-reversed rainbow trout. Theriogenology.

[103] Cabrita E., Robles V., Rebordinos L., Sarasquete C., Herráez M.P. (2005). Evaluation of DNA damage in rainbow trout (*Oncorhynchus mykiss*) and gilthead sea bream (*Sparus aurata*) cryopreserved sperm. Cryobiology.

[104] Dietrich G., Szpyrka A., Wojtczak M., Dobosz S., Goryczko K., Żakowski Ł., Ciereszko A., Dietrich M.A. (2005). Effects of UV irradiation and hydrogen peroxide on DNA fragmentation, motility and fertilizing ability of rainbow trout (Oncorhynchus mykiss) spermatozoa. Theriogenology.

[105] Pérez-Cerezales S., Martínez-Páramo S., Beirão J., Herráez M.P. (2010). Fertilization capacity with rainbow trout DNA-damaged sperm and embryo developmental success. Reproduction.

[106] Robles V., Cabrita E., Cuñado S., Herráez M.P. (2003). Sperm cryopreservation of sex-reversed rainbow trout (*Oncorhynchus mykiss*): Parameters that affect its ability for freezing. Aquaculture.

[107] Judycka S., Nynca J., Ciereszko A. (2019). Opportunities and challenges related to the implementation of sperm cryopreservation into breeding of salmonid fishes. Theriogenology.

[108] Bromage N., Porter M., Randall C. (2001). The environmental regulation of maturation in farmed finfish with special reference to the role of photoperiod and melatonin. Aquaculture.

[109] Labbe C., Maisse G. (1996). Influence of rainbow trout thermal acclimation on sperm cryopreservation: Relation to change in the lipid composition of the plasma membrane. Aquaculture.

[110] Fenkes M., Fitzpatrick J.L., Ozolina K., Shiels H.A., Nudds R.L. (2017). Sperm in hot water: Direct and indirect thermal challenges interact to impact on brown trout sperm quality. J. Exp. Biol..

[111] Wolf K. (1963). Physiological salines for fresh-water teleosts. Progress. Fish-Cult..

[112] Morisawa S., Morisawa M. (1988). Induction of potential for sperm motility by bicarbonate and pH in rainbow trout and chum salmon. J. Exp. Biol..

[113] Kobayashi T., Fushiki S., Ueno K. (2004). Improvement of sperm motility of sex-reversed male rainbow trout, Oncorhynchus mykiss, by incubation in high-ph artificial seminal plasma. Environ. Boil. Fishes.

[114] Itoh A., Inaba K., Ohtake H., Fujinoki M., Morisawa M. (2003). Characterization of a cAMP-dependent protein kinase catalytic subunit from rainbow trout spermatozoa. Biochem. Biophys. Res. Commun..

[115] Alavi S.M.H., Cosson J., Bondarenko O., Linhart O. (2019). Sperm motility in fishes: (III) diversity of regulatory signals from membrane to the axoneme. Theriogenology.

[116] Tresguerres M., Barott K.L., Barron M.E., Roa J.N. (2014). Established and potential physiological roles of bicarbonate-sensing soluble adenylyl cyclase (sAC) in aquatic animals. J. Exp. Biol..

[117] McNiven M., Gallant R., Richardson G. (1993). Fresh storage of rainbow trout (*Oncorhynchus mykiss*) semen using a non-aqueous medium. Aquaculture.

[118] Contreras P., Dumorné K., Ulloa-Rodríguez P., Merino O., Figueroa E., Farías J.G., Valdebenito I., Risopatrón J. (2019). Effects of short-term storage on sperm function in fish semen: A review. Rev. Aquac..

[119] Cabrita E., Robles V., Herraez P., Bobe J., Labbe C. (2009). Chilled storage of sperm and eggs. Methods in Reproductive Aquaculture, Marine and Freshwater Species.

[120] Cabrita E., Sarasquete C., Martínez-Páramo S., Robles V., Beirão J., Pérez-Cerezales S., Herráez M.P. (2010). Cryopreservation of fish sperm: Applications and perspectives. J. Appl. Ichthyol..

[121] McAndrew B.J., Rana K.L., Penman D.J., Muir J.F., Roberts R.J. (1993). Conservation and preservation of genetic variation in aquatic organisms. Recent Advances in Aquaculture.

[122] Lubzens E., Rothbard S., Hadani A. (1993). Cryopreservation and viability of spermatozoa from the ornamental Japanese carp. Isr. J. Aquac..

[123] Erdahl D.A., Graham F.F. (1980). Preservation of spermatozoa of brook trout and rainbow trout. CryoLetters.

[124] Nynca J., Judycka S., Liszewska E., Dobosz S., Ciereszko A. (2017). Standardization of spermatozoa concentration for cryopreservation of rainbow trout semen using a glucose-methanol extender. Aquaculture.

[125] Judycka S., Nynca J., Liszewska E., Dobosz S., Grudniewska J., Ciereszko A. (2018). Optimal sperm concentration in straws and final glucose concentration in extender are crucial for improving the cryopreservation protocol of salmonid spermatozoa. Aquaculture.

[126] Judycka S., Żarski D., Dietrich M.A., Palińska-Żarska K., Karol H., Ciereszko A. (2019). Standardized cryopreservation protocol of European perch (*Perca fluviatilis*) semen allows to obtain high fertilization rates with the use of frozen/thawed semen. Aquaculture.

[127] Horvath A., Labbé C., Jesensek D., Hoitsy G., Bernáth G., Kaczkó D., Bokor Z., Urbányi B. (2015). Post-thaw storage of sperm from various salmonid species. J. Appl. Ichthyol..

[128] Nynca J., Dietrich G., Dobosz S., Zalewski T., Ciereszko A. (2015). Effect of postthaw storage time and sperm-to-egg ratio on fertility of cryopreserved brook trout sperm. Theriogenology.

[129] Billard R. (1992). Reproduction in rainbow trout: Sex differentiation, dynamics of gametogenesis, biology and preservation of gametes. Aquaculture.

[130] Bailey J.L., Bilodeau J.F., Cormier N. (2000). Semen cryopreservation in domestic animals: A damaging and capacitating phenomenon. J. Androl..

[131] Watson P. (2000). The causes of reduced fertility with cryopreserved semen. Anim. Reprod. Sci..

[132] Li P., Hulak M., Koubek P., Sulc M., Dzyuba B., Boryshpolets S., Rodina M., Gela D., Maňásková P., Pěknicová J. (2010). Ice-age endurance: The effects of cryopreservation on proteins of sperm of common carp, *Cyprinus carpio* L.. Theriogenology.

[133] Guthrie H., Welch G. (2012). Effects of reactive oxygen species on sperm function. Theriogenology.

[134] Figueroa E., Merino O., Risopatrón J., Isachenko V., Sánchez R., Effer B., Isachenko E., Farias J., Valdebenito I. (2015). Effect of seminal plasma on Atlantic salmon (*Salmo salar*) sperm vitrification. Theriogenology.

[135] Merino O., Risopatrón J., Sanchez R., Isachenko E., Figueroa E., Valdebenito I., Isachenko V. (2011). Fish (*Oncorhynchus mykiss*) spermatozoa cryoprotectant-free vitrification: Stability of mitochondrion as criterion of effectiveness. Anim. Reprod. Sci..

[136] Merino O., Sánchez R., Risopatrón J., Isachenko V., Katkov I., Figueroa E., Valdebenito I., Mallmann P., Isachenko V. (2012). Cryoprotectant-free vitrification of fish (*Oncorhynchus mykiss*) spermatozoa: First report. Andrologia.

[137] Kása E., Lujić J., Marinović Z., Kollár T., Bernáth G., Bokor Z., Urbányi B., Lefler K.K., Jesenšek D., Horváth Á. (2018). Development of sperm vitrification protocols for two endangered salmonid species: The Adriatic grayling, Thymallus thymallus, and the marble trout, Salmo marmoratus. Fish Physiol. Biochem..

[138] Fahy G., Macfarlane D., Angell C., Meryman H. (1984). Vitrification as an approach to cryopreservation. Cryobiology.

[139] Truscott B., Idler D.R., Hoyle R.J., Freeman H.C. (1968). Sub-zero preservation of atlantic salmon sperm. J. Fish. Res. Board Can..

[140] Martínez-Páramo S., Horvath A., Labbé C., Zhang T., Robles V., Herráez P., Suquet M., Adams S., Viveiros A.T.M., Tiersch T.R. (2017). Cryobanking of aquatic species. Aquaculture.

[141] Dietrich M.A., Nynca J., Ciereszko A. (2019). Proteomic and metabolomic insights into the functions of the male reproductive system in fishes. Theriogenology.

[142] Leeds T.D., Vallejo R.L., Weber G.M., Gonzalez-Pena D., Silverstein J.T. (2016). Response to five generations of selection for growth performance traits in rainbow trout (*Oncorhynchus mykiss*). Aquaculture.

